# Anterior Cruciate Ligament Return to Sport after Injury Scale (ACL-RSI) Scores over Time After Anterior Cruciate Ligament Reconstruction: A Systematic Review with Meta-analysis

**DOI:** 10.1186/s40798-024-00712-w

**Published:** 2024-04-30

**Authors:** Timothy C. Sell, Ryan Zerega, Victoria King, Charles R. Reiter, Hailey Wrona, Garrett S. Bullock, Nilani Mills, Anu Räisänen, Leila Ledbetter, Gary S. Collins, Joanna Kvist, Stephanie R. Filbay, Justin M. Losciale

**Affiliations:** 1 Atrium Health Musculoskeletal Institute, Charlotte, NC USA; 2 Department of Physical Therapy, University of British Columbia, Vancouver, Canada; 3 Department of Orthopaedic Surgery, Wake Forest School of Medicine, Winston Salem, NC USA; 4 Centre for Sport, Exercise and Osteoarthritis Research Versus Arthritis, University of Oxford, Oxford, UK; 5 University of New South Wales, Sydney, NSW Australia; 6 Department of Physical Therapy Education-Oregon, College of Health Sciences-Northwest, Western University of Health Sciences, Oregon, USA; 7 Sport Injury Prevention Research Centre, Faculty of Kinesiology, University of Calgary, Calgary, Canada; 8 Medical Libraries, Duke School of Medicine, Durham, NC USA; 9 Centre for Statistics in Medicine, Nuffield Department of Orthopaedics, Rheumatology, and Musculoskeletal Sciences, University of Oxford, Oxford, UK; 10 Oxford University Hospitals NHS Foundation Trust, Oxford, UK; 11 Unit of Physiotherapy, Department of Health, Medicine, and Caring Medicine, University of Linkoping, Linköping, Sweden; 12 Department of Molecular Medicine and Surgery, Stockholm Sports Trauma Research Center, Karolinska Institute, Stockholm, Sweden; 13 Department of Physiotherapy, Centre for Health, Exercise and Sports Medicine, University of Melbourne, Melbourne, VIC Australia; 14 Arthritis Research Canada, Vancouver, Canada

**Keywords:** Psychological readiness, Return to sport, Knee, Athletes

## Abstract

**Background:**

Psychological readiness is an important consideration for athletes and clinicians when making return to sport decisions following anterior cruciate ligament reconstruction (ACLR). To improve our understanding of the extent of deficits in psychological readiness, a systematic review is necessary.

**Objective:**

To investigate psychological readiness (measured via the Anterior Cruciate Ligament-Return to Sport after Injury scale (ACL-RSI)) over time after ACL tear and understand if time between injury and surgery, age, and sex are associated with ACL-RSI scores.

**Methods:**

Seven databases were searched from the earliest date available to March 22, 2022. Articles reporting ACL-RSI scores after ACL tear were included. Risk of bias was assessed using the ROBINS-I, RoB-2, and RoBANS tools based on the study design. Evidence certainty was assessed for each analysis. Random-effects meta-analyses pooled ACL-RSI scores, stratified by time post-injury and based on treatment approach (i.e., early ACLR, delayed ACLR, and unclear approach).

**Results:**

A total of 83 studies were included in this review (78% high risk of bias). Evidence certainty was ‘weak’ or ‘limited’ for all analyses. Overall, ACL-RSI scores were higher at 3 to 6 months post-ACLR (mean = 61.5 [95% confidence interval (CI) 58.6, 64.4], I^2^ = 94%) compared to pre-ACLR (mean = 44.4 [95% CI 38.2, 50.7], I^2^ = 98%), remained relatively stable, until they reached the highest point 2 to 5 years after ACLR (mean = 70.7 [95% CI 63.0, 78.5], I^2^ = 98%). Meta-regression suggests shorter time from injury to surgery, male sex, and older age were associated with higher ACL-RSI scores only 3 to 6 months post-ACLR (heterogeneity explained R^2^ = 47.6%), and this reduced 1–2 years after ACLR (heterogeneity explained R^2^ = 27.0%).

**Conclusion:**

Psychological readiness to return to sport appears to improve early after ACL injury, with little subsequent improvement until ≥ 2-years after ACLR. Longer time from injury to surgery, female sex and older age might be negatively related to ACL-RSI scores 12–24 months after ACLR. Due to the weak evidence quality rating and the considerable importance of psychological readiness for long-term outcomes after ACL injury, there is an urgent need for well-designed studies that maximize internal validity and identify additional prognostic factors for psychological readiness at times critical for return to sport decisions.

*Registration*: Open Science Framework (OSF), https://osf.io/2tezs/.

**Supplementary Information:**

The online version contains supplementary material available at 10.1186/s40798-024-00712-w.

## Key Points

- Over 70% of studies measuring ACL-RSI scores were judged to have high risk of bias (i.e., low internal validity), leading to a certainty of evidence rating of weak for all analyses.
- ACL-RSI scores do not appear to increase during typical rehabilitation periods after anterior cruciate ligament reconstruction.
- Age, sex, and time from injury to surgery (i.e., treatment approach) appear prognostic for early ACL-RSI scores, but their prognostic ability diminishes for later ACL-RSI scores.

**Supplementary Information:**

The online version contains supplementary material available at 10.1186/s40798-024-00712-w.

## Background

Anterior cruciate ligament (ACL) tear is a severe injury common in sports that involve cutting, landing, and sharp deceleration [1–4]. Irrespective of treatment with surgery or not, ACL injury predisposes individuals to additional knee injuries [5], osteoarthritis [6], and can negatively affect participation in activities necessary for health and wellness [7–9]. Successful rehabilitation and return-to-sport (RTS) requires complex decisions that should include musculoskeletal, neuromuscular, physiological fitness, and movement assessments [10, 11]. Increasingly, clinicians are recognizing the significant impact that psychological factors, such as fear and confidence, have on successful RTS and re-injury risk [12–14].

The Anterior Cruciate Ligament-Return to Sport after Injury scale (ACL-RSI) was specifically designed to assess readiness to RTS following ACL reconstructive surgery (ACLR) [15]. The ACL-RSI includes 12 items about confidence and fear relative to performance, reinjury, and returning to pre-injury sport participation [15]. A shorter version (6 items) has also been developed [16]. Research investigating ACL-RSI scores after ACL injury has highlighted the importance of psychological readiness in an attempt to prevent a second ACL injury [17, 18]. In a cohort of 450 patients, individuals who met a chosen ACL-RSI threshold (i.e., 65/100) at 6-months after ACLR were more likely to RTS at 12-months [18]. However, younger athletes with lower ACL-RSI scores experienced more second ACL tears [19]. Thus, the ACL-RSI tool has become a key metric for clinicians when working with athletes after ACLR.

Due to the clinical importance of psychological readiness measured via the ACL-RSI, it is important to understand what factors (both modifiable and non-modifiable) may affect ACL-RSI scores. These factors may help clinicians understand who are the most vulnerable ACL-reconstructed patients [20]. For example, high levels of fear of reinjury have been found before undergoing a delayed ACL reconstruction but the impact of this fear on ACL-RSI scores is not known [17]. Male or female sex may also impact ACL-RSI scores and subsequent injury risk [21]. These previous findings along with the impact of surgical timing on recovery demonstrate the importance of understanding the relationships between sex [22], age [19], and ACL-RSI score changes following ACL surgery. Research on the ACL-RSI has grown considerably over the past 15-years, however the data have yet to be consolidated to describe ACL-RSI scores over time or describe how important are factors such as age, sex, and the time from injury to surgery. This information can provide data points that clinicians can compare their patients to, as well as inform future research on prognostic factors for poor psychological recovery. Therefore, the purpose of this systematic review was to describe ACL-RSI scores over time after ACL injury and investigate factors that may affect ACL-RSI scores including the time between injury and surgery, age, and biological sex.

## Methods

### Study Design

This systematic review with meta-analysis followed the Finding What Works in Health Care: Standards for Systematic Reviews Handbook [23] and is reported according to the updated (2020) Preferred Reporting Items for Systematic Reviews and Meta-Analyses (PRISMA) guidelines [24, 25]. This review was prospectively registered on the Open Science Framework (OSF), https://osf.io/2tezs/.

### Information Sources

The databases searched included Medline (Ovid), Embase (Elsevier), CINAHL Complete (EBSCOhost), Web of Science Core Collection (Clarivate), Scopus (Elsevier), Cochrane Central Register of Controlled Trials (Cochrane Library), and SPORTDiscus with Full Text (EBSCOhost).

### Search Strategy

The search was developed and conducted by a professional medical librarian (LL) with input from the authorship team, and included a mix of keywords and subject headings representing the exposure (i.e., 'anterior cruciate ligament injury') and outcomes (e.g. ‘psychological readiness’). Search database filters were used to remove publication types such as systematic reviews, case studies, conference abstracts, editorials, letters, comments, and animal-only studies as appropriate for each database. The original search was performed on July 17, 2020, with an updated search on March 22, 2022. Bibliographies of selected studies were hand searched to identify relevant articles not captured by the search strategy. The reference lists of the final included articles were reviewed and citation tracking in Web of Science (May 2021) was used to identify relevant studies and those studies were added for full text review. Complete reproducible search strategies, including date ranges and search filters, for all databases are described in “Additioanl file 1: Appendix 1”. After the search, all identified studies were uploaded into Covidence (Veritas Health Innovation, Melbourne, Australia), a software system for managing systematic reviews. Duplicates were removed automatically by the software.

### Eligibility Criteria

The outcome of interest for this review was the ACL-RSI score measured at any time after ACL injury [16]. Studies were eligible for inclusion if they: (1) assessed the ACL-RSI in ACL-injured individuals (first or second ACL-injury (ipsilateral or contralateral)) at any time point after injury; (2) were written in English. Our exclusion criteria consisted of studies that included participants with: (1) three or more concomitant ligament ruptures or knee dislocation of the involved knee; (2) a history of three of more ACL ruptures on the same knee; (3) any synthetic or enhanced ligament grafting for ACLR (e.g., Ligament Advanced Reinforcement System (LARS) ligament/GORE-TEX enhanced, Leeds-Keio); (4) systematic reviews, meta-analyses, qualitative studies, clinical commentaries, case reports, editorials, conference abstracts, or letters to the editor.

### Study Selection

Each reviewer underwent a calibration training exercise prior to screening, consisting of independent screening of title, abstract, and full text of five studies, followed by group discussion of inclusion and exclusion of articles. Following training, title and abstracts were screened using inclusion and exclusion criteria after being randomly assigned to two of the three different reviewers (RZ, VK, and HW). Following abstract screening, the same reviewers then performed blinded full-text review of articles in duplicate. Any conflicts were first discussed between both reviewers. If a consensus could not be reached another reviewer was utilized to determine final study eligibility.

### Data Extraction

Eligible articles were divided amongst five pairs of reviewers and data were extracted into customized Excel spread sheets by two independent reviewers. If consensus could not be reached for data extraction, a third reviewer resolved data discrepancies. If data were vague and further detail was needed, authors were contacted to provide clarification on three occasions, each at least two weeks a part. If authors did not respond following the third attempt, specific data were considered not reported. Data extraction included: authors, journal, year published, study design, sample size, cohort age, sex, body mass index, graft type (if surgical), time from injury to treatment, follow-up time points/length, injury/surgery history, concomitant injury and treatment, pre-injury activity level, RTS status, RTS definition, ACL injury treatment, and ACL-RSI scores (as reported by each study).

### Categorization of ACL Treatment Strategies, Activity Level, and Intention to Return to Sport

For the purposes of between group comparisons, we established prior definitions of ACL treatment strategies. Early ACLR was defined as ACLR within a mean time of 6 months from ACL injury without trialing exercise therapy or following a period of “pre-habilitation” with the intention of undergoing surgery on completion. Delayed ACLR was defined as ACLR following a trial of management with exercise therapy (i.e., rehabilitation alone) or ACLR ≥ 6 months after injury, on average. Patients may have ‘crossed-over’ to ACL surgery for several reasons including episodes of functional knee instability, patient choice, surgeon recommendations, or inability to meet strength/functional milestones. We initially separated this group out to categorize studies with and without known exercise therapy before delayed ACLR, however due to a low number of studies we collapsed this to one category for analysis. Studies were classified as ‘ACL-repair’ if surgical repair of the native ligament was performed regardless of timing. Studies were classified as ‘rehabilitation only’ if a known exercise therapy was reported and surgical management did not occur. Due to limited information and lack of clarification, we made the post-hoc decision to include an ‘unclear’ category where the ACL treatment strategy (i.e., early or late) was unknown and could not be clarified. All unclear studies included participants who underwent ACLR.

Tools used to describe pre-injury activity level were heterogenous across studies, therefore, we assigned an activity level grade of recreational, competitive, elite, or unspecified based on the information within the study for descriptive purposes. Recreational was considered Tegner Activity Scale < 7 [26], descriptive activity participation of < 4 h/week, or International Knee Documentation Committee (IKDC) L3 activities [27]. Competitive was considered Tegner Activity Scale 7–9 [26], ≥ 4 h/week of activity and IKDC L1 or L2 activities [27]. Elite was only considered for Tegner Activity Scale 10 [26] or when there were clear indications the athletes played at a university, national, international or professional level. When a study did not provide enough information, activity level was deemed unspecified.

Commonly, individuals who are managed non-operatively with exercise-therapy only after an ACL injury receive a recommendation to adjust their activity level (avoid cutting/pivoting/impact sports) or not attempt RTS at their prior level. This can create an inherent selection bias when the ACL-RSI is the outcome because certain individuals are now less likely to have high readiness to RTS because they are no longer attempting RTS. To facilitate a sensitivity analysis to account for this potential bias, we defined ‘intention to RTS’ to be present in study design if it was clearly specified in the study selection criteria or when the proportion of participants who returned (i.e., RTS%) was > 50%. A similar definition of ‘intention to RTS’ has been used in a recent meta-analysis [28], and RTS rates can be as low as 55% (95% CI; 46%, 63%) for competitive athletes [29].

### Risk of Bias and Evidence Synthesis

Risk of bias (RoB) was assessed using domain-based RoB tools specific to study design, including the Cochrane Risk of Bias tool for randomized trials (RoB 2), Risk of Bias tool In Non-randomized Studies of Interventions (ROBINS-I), and the Risk of Bias Assessment tool for Nonrandomized Studies (RoBANS) [30–32]. Six independent reviewers assessed each study for RoB. If consensus could not be reached, a third reviewer resolved discrepancies. The strength of the evidence for pooled ACL-RSI scores per timepoint was derived based on RoB judgement of the individual studies and amount of evidence according to methods adapted from Teirlinck et al. [33] Specifically:

- Strong evidence: Data are provided by ≥ two studies in which 100% of the studies have a low risk of bias judgement in all assessed RoB domains.
- Moderate evidence: Data are provided by two studies in which > 25% of the studies have a moderate, high, or unclear risk of bias in ≤ one assessed RoB domain.
- Weak evidence: Data are provided by ≥ two studies in which > 25% of the studies have a moderate, high, or unclear risk of bias in ≥ two assessed RoB domains.
- Limited evidence: Data are provided by one study irrespective of RoB judgement.

### Deviation from Protocol

The original a priori protocol included the Tampa Scale of Kinesiophobia (TSK), Knee-Self Efficacy Scale (KSES), Fear Avoidance Beliefs Questionnaire (FABQ), ACL-RSI and ACL quality of life questionnaire outcome measures. Due to the large number of eligible studies reporting these outcomes (149 studies), findings for the other measures have been reported in a separate manuscript to allow comprehensive reporting of ACL-RSI results and a more complete discussion [34]. This decision was made after eligibility screening was complete and before data extraction began. Further, the protocol was designed to allow an additional meta-analysis using individual participant data (IPD) if IPD were received from at least 50% of studies. However, this threshold was not met, so only an aggregate meta-analysis was performed.

### Statistical Analyses

Aggregated data were summarised using counts (percentages) and medians (ranges). If a study reported an eligible outcome for two subgroups, the subgroup outcomes were combined using The Cochrane Handbook for Systematic Reviews of Interventions formula to obtain the mean and standard deviation estimates [35]. If a study only reported 95% confidence intervals without standard deviations, standard deviations were estimated using the square root of the sample size and corresponding t scores [35]. If a study reported median and quantiles, minimum and maximum, or interquartile range, outcome data were converted to mean and standard deviation through the method by McGrath et al. [36] Time units (e.g., days, months, and years) were converted to the same unit for analyses (days).

Pooled ACL-RSI scores were obtained using aggregate Der Simonian and Laird random effect meta-analysis models with inverse variance weighting, stratified by time since ACL injury (i.e., pre-operative or prior to rehabilitation, 3 to 6 months post-ACLR, 7 to 12 months post-ACLR, 1 to 2 years post-ACLR, and 2 to 5 years post-ACLR). Heterogeneity was assessed through overall Tau score and I^2^ (heterogeneous: graded as I^2^ > 50%), with a priori alpha of p < 0.10. Meta-regressions were performed to investigate the explanation of variance of different key factors. We sought to use ACL treatment group (expressed as days from injury to treatment), percent female, mean age, and percent of participants with concomitant knee injuries in the meta-regression because these factors could potentially influence someone’s readiness for sport. However, due to missing data, using these four factors resulted in an underpowered primary meta-regression (n = 6) and an unreliable result. Thus, we removed the percentage of participants with concomitant injuries to focus on participant characteristics and the core variable of interest, time from injury to treatment. This increased the number of available studies to 9 (3 to 6 months) and 11 (7 to 12 months). Funnel plots were generated to assess for publication bias. To understand the stability of our findings that may have been introduced by an inherent selection bias, we performed a sensitivity analysis that restricted the primary meta-analysis to include studies where we judged there to be an intention for participants to RTS. All analyses were performed in R version R Core Team (2021). R: A language and environment for statistical computing. R Foundation for Statistical Computing, Vienna, Austria. URL https://www.R-project.org/. The *meta* package was used for all meta-analyses.

## Results

### Literature Search

**Fig. 1:**
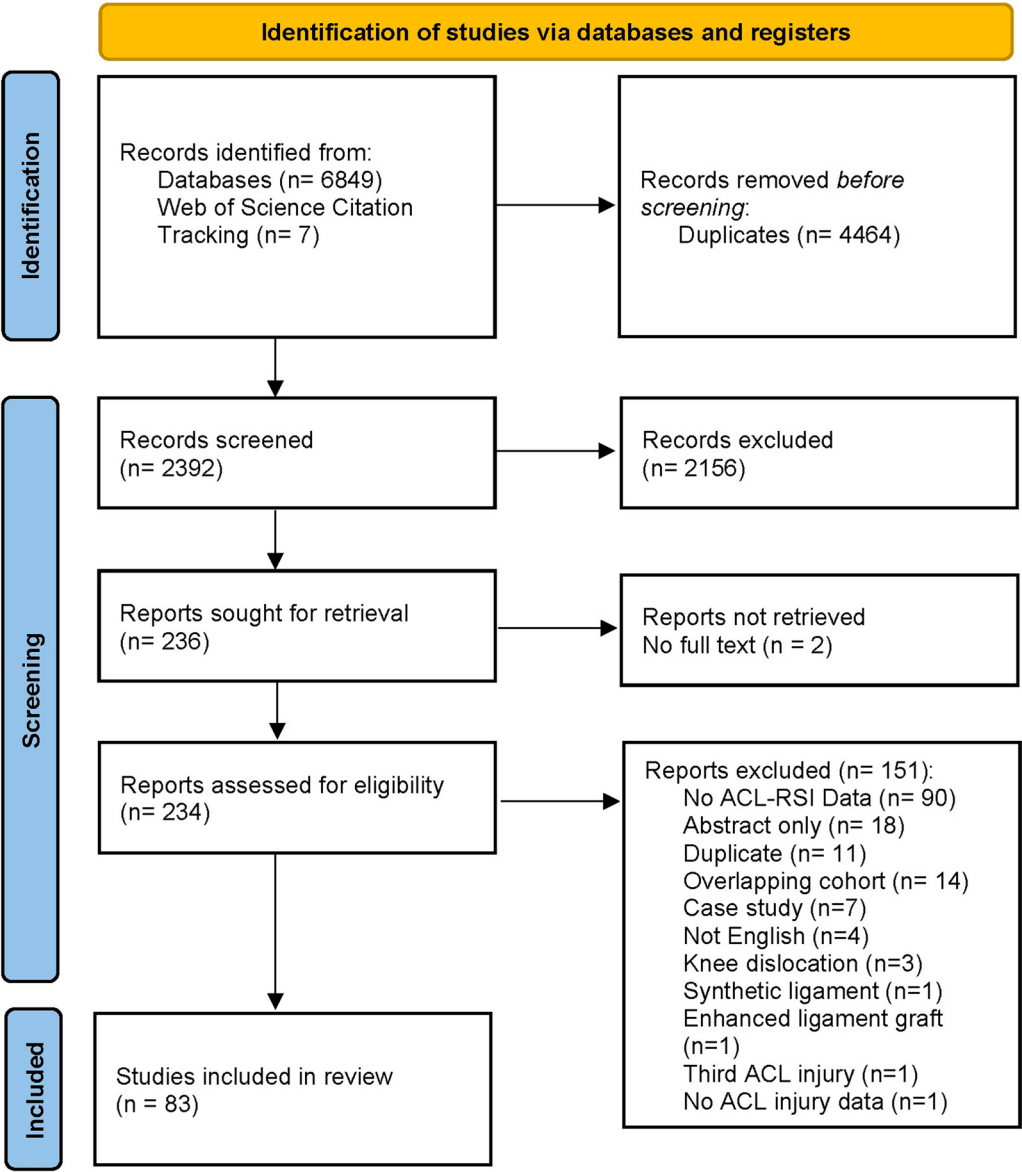
Preferred Reporting Items for Systematic Reviews and Meta-Analyses (PRISMA) Flowchart

The systematic search yielded 6856 potentially eligible studies, with 7 of those obtained from citation tracking search. A total of 2392 articles underwent screening after duplicate removal, with 234 undergoing full-text review. In total, 83 studies were deemed eligible for this review (Fig. 1) [13, 15, 16, 19, 21, 37–115]. Forty-eight corresponding authors were contacted via email to clarify data for 61 studies. We received clarification regarding 21 studies (34.4%). The authors contacted and the outcomes of data clarification are reported in “Additioanl file 1: Appendix 2”.

### Study Characteristics and Demographics

Study characteristics and participant demographics are provided in Table 1. Five studies included mutually exclusive groups that were judged to have received a different treatment strategy. Those five studies were then subdivided and treated as separate studies for the meta-analyses, and thus there were 88 studies in the meta-analysis (from the 83 original, included studies). Thirty-four studies (out of 88 studies included in the meta-analysis, 38.6%) included participants managed with early ACLR, 15 (17.0%) studies included participants managed with delayed ACLR, 2 (2.3%) studies included participants managed with rehabilitation only, and in 37 (42.1%) studies the treatment strategy was unclear.

**Table 1:** Study characteristics

ACL Injury Treatment Category^a^	References	Study Design^b^	Sample Size (n), % F	Age at time of ACL treatment (yr)	Standardized Pre-injury Activity Level^c^	Intent to RTS^d^	Time from Injury to Treatment
Early ACLR	Ardern et al. [40]	CC	ALL = 122, 37.7% RTSSS = 47 NRTSSS = 67	ALL = NR RTSSS = 27.5 ± 8.3 NRTSSS = 28.9 ± 8.5	Comp	Y	3 mo^g^
	Burland et al. [46]	CC	All = 21, 52.4% LPD = 11 HPD = 10	20.9 ± 2.9^h^	U	ALL = Y LPD = Y HPD = Y	80.1 ± 163.6 d^i^
	Burland et al. [47]	CC	ALL = 50, 46% RTS = 34, 38.2% NRTS = 16, 62.5%	15.9 ± 1.8	U	ALL = Y RTS = Y NRTS = N	> 6 mo^i^
	Culvenor et al. [50]	CC	ALL = 110, 30.9% PFP = 33, 36.4% NPFP = 77, 28.6%	ALL = NR PFP = 34 ± 9 NPFP = 29 ± 8	ALL = Comp PFP = Comp NPFP = Comp	All = Y PFP = Y NPFP = Y	ALL = NR PFP = 3.1 (3.3) mo NPFP = 3.6 (8.7) mo
	Ebert et al. [52]	C-S	50, 32%	28.3 ± 9.1^k^	U	N	10.4 ± 11.9 wk
	Ehlinger et al. [53]	PC	Surgical = 228, 59%	54.8 ± 4.3	Rec	Y	23.6 ± 59 d
	Faltstrom et al. [55]	CC	ALL = 44, 100% RTS = 29, 100% NRTS = 15, 100%	NR	NR	ALL = Y RTS = Y NRTS = N	0–90 days^j^
	Fones et al. [58]	CC	ALL = 74, 62.2% RTSS = 54, 59.3% NRTSS = 20, 70%	ALL = 15.9 ± 1.5 RTSS = 15.8 ± 1.4 NRTSS = 16 ± 1.6	ALL = Comp RTSS = Comp NRTSS = Comp	ALL = Y RTSS = Y NRTSS = N	ALL = NR RTSS = 51.2 ± 33.3 d NRTSS = 91 ± 84.9 d
	Johnston et al. [67]	RC	ALL = 111, 21.6% QT = 37, 21.6% HT = 74, 21.6%	ALL = NR QT = 20 [15–34] HT = 20.5 [15–32]	ALL = Comp QT = Comp HT = Comp	ALL = Y QT = Y HT = Y	ALL = NR QT = 49 [11–482] d HT = 59 [16–2143] d
	Kitaguchi et al. [69]	CC	ALL = 124, 59.7% RTSSS = 101, 58.4% NRTSSS = 23, 65.2%	ALL = 17 ± 2.7 RTSSS = 16.9 ± 2.5 NRTSSS = 17.7 ± 3.1	ALL = Comp RTSSS = Comp NRTSSS = Comp	ALL = Y RTSSS = Y NRTSSS = Y	ALL = 3.3 ± 4.2 mo RTSSS = 3.2 ± 4.5 mo NRTSSS = 3.6 ± 2.7 mo
	Klasan et al. [70]	CS	123, 42.3%	30 [18–60]	Comp	Y	5.6 ± 14.3 mo^i^
	Kostyun et al. [71]	CS	51, 100%	15.3 ± 1.4	U	U	2.7 ± 4 mo
	Langford et al. [73]	CC	ALL = 87, 36.8% RTSSS = 44 NRTSSS = 43	27.5 ± 5.7^k^	ALL = Comp RTSSS = Comp NRTSSS = Comp	ALL = Y RTSSS = Y NRTSSS = Y	18, 2–216 wk
	Lepley et al. [75]	CS	20, 55%	20.9 ± 4.4	U	U	Pre-ACLR TP = 37.1 ± 15.3 d post-injury
	Lisee et al. [77]	C-S	25, 52%	M = 16.2 ± 1.6^h^ F = 16.4 ± 1.3^h^	ALL = U M = Elite F = Comp	ALL = Y M = Y F = Y	M = 29.4 ± 13.1 d F = 27.3 ± 19.8 d
	Martini et al. [78]	CS	37, 35.1%	27.2 ± 9.2^h^	Comp	U	11 (8–22) wk^i^
	Meierbachtol et al. [80]	C-S	33, 54.5%	18.1 ± 4.8	Comp	Y	3–6 wk^i^
	Meierbachtol et al. [81]	IS	58, 63.8%	21.2 ± 7.8	U	Y	3–6 wk^i^
	Monaco et al. [82]	CC	115, 27.8%	25.6 ± 8.2^h^	Rec	Y	23.1 ± 8.4 d
	Muller et al. [83]	IS	RTSSS = 31, 38.7%	31.4 ± 10.3	Comp	Y	13.3 ± 19.2 wk
	Nagelli et al. [84]	C-S	18, 50%	20 ± 7.4^h^	U	U	55.2 ± 56.3 (missing for n = 1)^i^
	Ohji et al. [85]	CC	ALL = 39, 43.6% NRTSSS = 16, 31.3% RTSSS = 23, 52.2%	NRTSSS = 23 (20.3)^h^ RTSSS = 20 (4)^h^	ALL = Comp NRTSSS = Comp RTSSS = Comp	ALL = Y NRTSSS = Y RTSSS = Y	NRTSSS = 68.5 (41) d RTSSS = 81 (100) d
	Ohji et al. [86]	CC	ALL = 50, 54% < 56 = 17, 41.2% ≥ 56 = 33, 60.6%	ALL = 20.5 (11)^h^ < 56 = 20 (11)^h^ ≥ 56 = 21 (1)^h^	All = Comp < 56 = Comp ≥ 56 = comp	U	ALL = 58 (61) d < 56 = 71.5 (143) d ≥ 56 = 46.5 (47) d
	Panisset et al. [87]	PC	ALL = 358 ≥ 50 yr at ACLR = 228, 59% ≤ 40 yr at ACLR = 130, 35%	ALL = NR ≥ 50 yr at ACLR = 54.8, 50–71.6 ≤ 40 yr at ACLR = 26.7, 13.7–40	U	ALL = Y ≥ 50 yr at ACLR = Y ≤ 40 yr at ACLR = Y	ALL = NR ≥ 50 yr at ACLR = 23.6 wk ≤ 40 yr at ACLR = 8.7 wk
	Peebles et al. [88]	C-S	38, 42.1%	16.3 ± 1.9^h^	U	U	42.7 ± 35.6 d^i^
	Sala-Barat et al. [97]	C-S	ALL = 114, 14.9% IRTS = 92 NIRTS = 22	21.8 ± 5.2^h^	ALL = Comp IRTS = Comp NIRTS = Comp	ALL = Y IRTS = Y NIRTS = N	< 3 mo = 50.9% 3–6 mo = 32.4% > 6 mo = 16.7%
	Sanborn et al. [100]	RCT	35, 54.3%	17 (15–23)^h^	Comp	U	39 (33–43) d
	Toale et al. [104]	CC	RTS = 1140	23.6 ± 7	U	Y	4.1 ± 12.7 mo^i^
	Webster et al. [16]	CC	535, 35.1%	26.8 ± 9 yr (at 12-mo post-ACLR)	U	ALL = Y NRTS = N RTT = Y RTSS = Y RTSSS = Y	~ 70% w/in 6 mo^i^
	Webster et al. [15]	C-S	220, 43.6%	29.2 ± 9.7^h^	ALL = Comp RTS = Comp NRTS = Comp AG NIRTS = Comp IRTS = Comp RTT = Comp RTSSS = Comp	ALL = Y RTS = Y NRTS = Y AG NIRTS = Y IRTS = Y RTT = Y RTSSS = Y	~ 75% w/in 6 mo^i^
	Webster et al. [13]	CC	ALL = 222, 40.5% RTSSS = 135, 42.2% NRTSSS = 87, 37.9%	RTSSS = 25 ± 9^h^ NRTSSS = 27 ± 9^h^	ALL = Comp RTSSS = U NRTSSS = U	ALL = Y RTSSS = Y NRTSSS = Y	83% w/in 6 mo^i^
	Webster et al. [109]	CC	ALL = 635, 38.7% RTSSS = 158, 25.9% NRTSSS = 477, 43%	ALL = 28 ± 10^h^ RTSSS = 26 ± 8^h^ NRTSSS = 29 ± 11^h^	ALL = Rec RTSSS = Comp NRTSSS = Rec	ALL = N RTSSS = Y NRTSSS = N	77% w/in 6 mo^i^
	Winkler et al. [112]	RC	ALL = 102, 48% SGF = 58, 50% MGF = 44, 45.5%	ALL At 1st R-ACLR = 24 [13–58] SGF At ACLR = 17.5 [12–53] At 1st R-ACLR = 22.5 [13–58] MGF At ACLR = 17 [12–34] At 1st R-ACLR = 24 [15–49]	U	U	5 ± 9 mo^i^
	Vermeijden et al. [107]	CS	96, 45.8%	35.3 ± 12.3^h^	Rec	U	31 [19–94] d
Delayed ACLR without prior exercise therapy	Ardern et al. [29]	CC	ALL = 164, 39.6% RTSS = 66, 39.4% NRTSS = 98, 39.8%	26 [18–45]^h^	ALL = Comp RTSS = Comp NRTSS = Comp	ALL = N RTSS = Y NRTSS = N	600 ± 878 d (missing for n = 7)^i^
	Ardern et al. [39]	CC	(TP1) ALL = 178, 35.4% RTSSS = 56, 30.4% NRTSSS = 122, 37.7% (TP2) ALL = 117, 38.4% RTSSS = 34 NRTSSS = 83	27.3 ± 8.7	ALL = Comp RTSSS = Comp NRTSSS = Comp	ALL = Y RTSSS = Y NRTSSS = Y	29.4 ± 65.9 wk
	Beischer et al. [41]	CC	(TP1) ALL = 384, 50% 15–20 yr = 174 21–30 yr = 210 AG NRMF = 236, 53.8% RMF = 66, 42.4% (TP2) ALL = 271, 52.4% 15–20 yr = 123 21–30 yr = 148 AG NRMF = 146, 55.5% RMF = 44, 36.4%	(TP1) ALL = 22.1 ± 4.5 (TP2) ALL = 22.2 ± 4.6	ALL = Comp 15–20 yr = Comp 21–30 yr = Comp NRMF = Comp RMF = Comp	ALL = Y 15–20 yr = Y 21–30 yr = Y NRMF = N RMF = Y	(TP1) ALL = 8.7 ± 13.7 mo (TP2) ALL = 7.7 ± 12.6 mo
	Faleide et al. [54]	CC	ALL = 103, 46.6% RTSSS = 43 NRTSSS = 60	28.7 ± 10	ALL = Comp RTSSS = Comp NRTSSS = Comp	ALL = Y RTSSS = Y NRTSSS = Y	8 (11) mo
	Fayard et al. [57]	CS	398, 54.3%	54 ± 4.1	U	Y	28.4, 0.3–530.8 wk
	Hart et al. [64]	C-S	118, 35.6%	31 ± 9^h^	Comp	N	14.4 ± 40.2 mo^i^
	Kostyun et al. [71]	CS	M = 42, 0%	15.1 ± 1.5	U	U	6.6, 5–11.2
	Kvist et al. [72]	C-S	ALL = 182, 41.8% RTSS = 73 NRTSS = 101 AG RTSSS = 48 NRTSSS = 124 Retest = 48, 47.9%	ALL = 28.5 ± 8.2^h^ Retest = 29 ± 9.5^h^	ALL = Comp RTSS = U NRTSS = U RTSSS = U NRTSSS = U Retest = NA	ALL = N RTSS = Y NRTSS = N RTSSS = Y NRTSSS = N Retest = U	629.4 ± 951.7 d (missing for n = 5)^i^
	Muller et al. [83]	IS	NRTSSS = 8, 75%	33 ± 10.5	Comp	Y	30.6 ± 39.3 wk
	Pioger et al. [90]	RC	ALL = 409, 23.5% ≥ 12 mm = 247, 16.2% < 12 mm = 111, 29.8%	28.6 ± 8.8 (at 69.6 ± 29 mo^o^)	U	U	15.7 ± 34.6 mo
	Piussi et al. [91]	RC	ALL = 328, 63.4% LSI ≥ 90% = 96, 57.3% LSI ≤ 85% = 56, 64.3% LSI < 85% > 90% = 176, 66.5%	26 ± 9.9	ALL = Comp LSI ≥ 90% = Comp LSI ≤ 85% = Comp LSI < 85% > 90% = Comp	U	373 ± 832.3 d
	Rayes et al. [93]	PC	ALL = 72, 13.9% BPTB = 36, 13.9% HT = 36, 13.9%	(TP2) BPTB: 22.6 ± 4.7 HT: 24 ± 4.9	U	Y	BPTB: 5.9 ± 10.9 mo HT: 6.7 ± 10.9 mo
	Toale et al. [104]	CC	NRTS = 222	27.2 ± 7.5	U	Y	8.3 ± 26.3 mo^i^
Delayed ACLR	Cusumano et al. [51]	RCT	ALL = 50, 10% Auto = 25, 12% Allo = 25, 8%	ALL = NR Auto = 27 ± 7^k^ Allo = 30 ± 6^k^	U	Y	ALL = 359 ± 686 d^i^ Auto = 520 ± 935 d^i^ Allo = 199 ± 110 d^i^
	Toanen et al. [105]	CS	12, 58.3%	61 ± 1.4	U	Y	11.5, 6–18 mo
Rehabilitation Only	Ehlinger et al. [53]	PC	Non-surgical = 92, 62%	59.9 ± 6.6	Rec	U	NA
	Fayard et al. [56]	CC	ALL = 41, 41.5% Partial ACL Tear = 25 Complete ACL Tear = 16	NR	Comp	N	60, 2–271 d (to diagnosis)
Unclear	Alzrani et al. [37]	CC	60, 0%	31.4 ± 7.2^h^	U	ALL = Y RTS = Y NRTS = Y	NR
	Betsch et al. [42]	CS	113, 37%	28.1 ± 8.1^h^	Comp	Y	NR
	Blakeney et al. [43]	CS	ACLR = 371, 22.6% Retest = 33, 24.2%	ACLR = 28 ± 9.9^h^ Retest = 22.9 ± 6.2^h^	ACLR = Rec Retest = Rec	U	NR
	Bohu et al. [44]	C-S	91, 27.5%	31.7 ± 8.1^h^	ALL = Rec RTSS = U NRTSS = U	ALL = Y RTSS = Y NRTSS = N	NR
	Bortone et al. [45]	CC	ALL = 30, 0% R = 15, 0% CP = 15, 0%	ALL = 26.9 ± 5.7^k^ R = 25.2 ± 7.6^k^ CP = 28.6 ± 1.9^k^	Comp	Y	NR
	Byrne et al. [48]	PC	ALL = 313, 0% C = 155, 0% MS = 128, 0% MR = 30, 0%	C = 23.3 ± 5 MS = 22 ± 5.4 MR = 23.8 ± 5.6	U	Y	NR
	Chen et al. [49]	CC	112, 26.8%	26.6 ± 7.6^h^	U	Y	NR
	Faltstrom et al. [55]	CC	ALL = 131, 100% RTSS = 62, 100% NRTSS = 69, 100%	NR	NR	ALL = N RTSS = Y NRTSS = N	91–365 days^j^
	Gokeler et al. [59]	C-S	28, 21.4%	25.5 ± 8.3^h^	Comp	Y	NR
	Goto et al. [60]	RC	ALL = 73, 100% S = 22, 100% L = 51, 100%	ALL = 33.8 ± 11.4^h^ S = 35.4 ± 10.9^h^ L = 33.1 ± 11.7^h^	U	U	NR
	Ha et al. [61]	C-S	129, 20.9%	28.3, 16-58^h^	U	U	NR
	Harput et al. [62]	C-S	90, 0%	28.5 ± 8.2^h^	U	Y	NR
	Harput et al. [63]	C-S	93, 5.4%	28.7 ± 8.6^h^	Comp	Y	NR
	Hirohata et al. [65]	C-S	93, 54.8%	25.8 ± 10.2^g^	U	Y	NR
	Jia et al. [66]	C-S	122, 41.8%	34 ± 13^k^	U	N	< 3 mo = 18.9% 3–12 mo = 63.9% > 12 mo = 17.2%
	Kim et al. [68]	CC	ALL = 85, 45.9% RTS = 67, 40.3% NRTS = 18, 66.7%	ALL = 29.2 ± 12.2^h^ RTS = 28.7 ± 11.8^h^ NRTS = 30.9 ± 13.4^h^	ALL = Rec RTS = Rec NRTS = Rec	ALL = Y RTS = Y NRTS = U	NR
	Kuenze et al. [21]	C-S	90, 50%	ALL = NR F = 18.8 ± 2.8^h^ M = 18.7 ± 2.7^h^	ALL = Comp F = Comp M = Comp	ALL = N F = N M = N	NR
	Lee et al. [74]	CC	ALL = 98, 26.5% A = 49, 26.5% B = 49, 26.5%	ALL = NR A = 17.4 ± 1.3^h^ B = 32.3 ± 4.9^h^	ALL = Rec A = Rec B = Rec	ALL = Y A = Y B = Y	NR
	Ling et al. [76]	RCT	ALL = 30, 60% Int = 15, 60% C = 15, 60%	ALL = 16, 12-20^h^ Int = 16, 13-18^h^ C = 16, 12-20^h^	ALL = Comp Int = Comp C = Comp	ALL = Yes C = NR Int = Yes	NR
	McPherson et al. [19]	CC	ALL = 329, 35.9% ≤ 20 yr = 132, 42.4% > 20 yr = 197, 31.5% AG NInj = 272 Injured at TP1 = 57 Injured at TP2 = 52	ALL = 25.3 ± 8.7 ≤ 20 yr = 17.7 ± 2.0 > 20 yr = 30.4 ± 7.7	U	ALL = Y ≤ 20 yr = Y > 20 yr = Y Inj = Y NInj = Y	NR
	Phelan et al. [89]	CC	ACLR = 499, 0%	24.3^h,h^	Comp	Y	NR
	Presley et al. [92]	CC	ALL = 92, 41.3% IS = 49, 42.9% OSI = 43, 39.5%	ALL = NR ISI = 25.5 ± 10.8^h^ OSI = 26.4 ± 13^h^	ALL = Comp ISI = Comp OSI = Comp	ALL = Y ISI = Y OSI = Y	NR
	Rhim et al. [94]	RCT	ALL = 32, 15.6% Int = 10, 10% P = 11, 27.3% C = 11, 9.1%	ALL = NR Int = 27.1 ± 9.5^k^ P = 32.2 ± 12.5^k^ C = 26.8 ± − 7.9^k^	U	U	NR
	Rosso et al. [95]	CS	176, 19.9%^s^	29.5 ± 9.6^k^	U	Y	NR
	Sadeqi et al. [96]	CC	681, 31.4%	30.2 ± 9.5	Rec	ALL = Y RTSS = Y NRTSS = N RTSSS = Y NRTSSS = N	NR
	Salatkaite et al. [98]	C-S	ALL = 65, 40% 6 mo = 43 12 mo = 22 AG RTSSS = 43 NRTSSS = 22	ALL = 25.6 ± 6.8^h^ 6 mo = 25.3 ± 6.7^h^ 12 mo = 26 ± 7^h^	ALL = U 6 mo = Rec 12 mo = Comp RTSSS = U NRTSSS = U	ALL = Y 6 mo = U 12 mo = U RTSSS = Y NRTSSS = N	NR
	Salatkaite et al. [99]	CC	ALL = 81, 37% LSI > 90% = 30, 33.3% LSI ≤ 90% = 51, 39.2%	ALL = NR LSI > 90% = 22.3 ± 5.3^h^ LSI ≤ 90% = 24.7 ± 7.5^h^	U	U	NR
	Slagers et al. [101]	CS	150, 44%	29.2 ± 10.8^k^	U	Y	NR
	Slagers et al. [102]	CS	70, 38.6%	27.5 ± 10.2	Comp	U	NR
	Thiebat et al. [103]	CC	130, 25.4%	31.2 ± 9.9^k^	Rec	Y	NR
	Tortoli et al. [106]	CC	129, 27%	28 ± 9^k^	U	Y	NR
	Webster et al. [108]	CS	ALL = 441, 41.7% M = 257, 0% F = 184, 100%	24.6 ± 7.4	Comp	Y	NR
	Welling et al. [110]	CC	62, 27.4%	24.2 ± 6.2^h^	U	Y	NR
	Willson et al. [111]	CS	23, 26.1%	13 ± 1.4	Comp	Y	NR
	Zanovello et al. [113]	CS	24, 20.8%	31.9 ± 11.2 (at R-ACLR)	Comp	Y	NR
	Zarzycki et al. [115]	C-S	ALL = 79, 49.4% Low = 19, 47.4% Mid = 40, 55% High = 20, 40%	ALL = NR Low = 22.3 ± 6.5^h^ Mid = 20.7 ± 7.7^h^ High = 21.0 ± 8.7^h^	U	U	NR
	Zarzycki et al. [114]	CC	ALL = 66, 50% Re = 39, 53.8% Non = 27, 44.4%	ALL = NR Re = 20.7 ± 7.6^h^ Non = 23 ± 8.8^h^	ALL = Comp Re = Comp Non = Comp	ALL = Y Re = Y Non = Y	NR

ACL Injury Treatment Category^a^	Proportion with Concomitant Injuries (%)^e^	Baseline for ACL-RSI^f^	TP1 ACL-RSI Results	TP2 for ACL-RSI^f^	TP2 ACL-RSI Results	TP3 for ACL-RSI^f^	TP3 ACL-RSI Results
Early ACLR	M = 54.1%	1 yr	46.3 (27) (missing for n = 20)	NA	NA	NA	NA
	NR	3.7 ± 3 yr	LPD = 88.8^j^ HPD = 59.2^j^	NA	NA	NA	NA
	M = 50%	3 mo	ALL = 64.2 ± 20.5 RTS = 66.4 ± 19.1 NRTS = 60.5 ± 22.9	6 mo	ALL = 74.1 ± 19.3 RTS = 76.7 ± 16.5 NRTS = 68.4 ± 24	NA	NA
	M = 40.9% PFCL = 10.9%	12.8 ± 0.8 mo	ALL = NR PFP = 43.8 ± 20.8 NPFP = 57.6 ± 20.3	NA	NA	NA	NA
	MR = 28% MS = 20%	10.2 ± 1.4 mo	61.7 ± 24.1	NA	NA	NA	NA
	M = 68% C = 76%	Pre-op	25 ± 20.2	14.2mo	70.8 ± 19.7	NA	NA
	NR	18 mo^g^	ALL = NR RTS = 6.9 ± 2^m^ NRTS = 4.5 ± 2.5^m^	NA	NA	NA	NA
	M = 40.5%	4 ± 2 yr	ALL = NR RTSS = 81.6 ± 20.4 NRTSS = 52.7 ± 26.7	NA	NA	NA	NA
	QT MM = 21.6% LM = 29.7% LM & MM = 2.7% HT MM = 16.2% LM = 20.3% LM & MM = 16.2%	6 mo	ALL = NR QT = 58.6 ± 23.7 HT = 58.2 ± 19.7	NA	NA	NA	NA
	ALL = 62.1% MM = 19.4% LM = 33.9% CL = 8.9% RTS MM = 19.8% LM = 33.9% CL = 8.9% NRTS MM = 17.4% LM = 30.4% CL = 4.4%	6 mo	ALL = 59.8 ± 19.6 RTSSS = 63.4 ± 18.7 NRTSSS = 43.7 ± 15.4	NA	NA	NA	NA
	M = 48% Ch = 7.3%	9 mo	Short (6-item) 51.3 ± 23.1^i^	NA	NA	NA	NA
	NR	0.8, 0.1–3.2 mo pre-ACLR	37.1 ± 25.1	3.1, 2.5–4.2 mo	54.1 ± 19.6	6.6, 5–11.2 mo	72.8 ± 19.5
	NR	3 mo	ALL = 55.7 ± 16.9 RTSSS = 60.1 ± 16.5 NRTSSS = 51.3 ± 16.2	6 mo	ALL = 57.6 ± 17.8 RTSSS = 63.2 ± 17.2 NRTSSS = 51.8 ± 16.8	12 mo	ALL = 65.4 ± 18.5 RTSSS = 72.1 ± 16.3 NRTSSS = 58.6 ± 18.3
	M = 65%	28.3 ± 2.9 wk	67.2 ± 28.1	NA	NA	NA	NA
	NR	ALL = 6.3 mo M = 6.2 ± 1.2 mo F = 6.4 ± 1.3 mo	M = 74.9 ± 19.7 F = 77.6 ± 10.8	NA	NA	NA	NA
	M = 29.8%	6 mo	58.8 ± 19.8	NA	NA	NA	NA
	NR	24.9 ± 3.1 wk	65.1 ± 18.1	Post-5 wk RTS training	80.1 ± 13.7	NA	NA
	NR	8.1 ± 1.8 mo	60.1 ± 19.3	40.5 ± 1.6 d^n^	77.9 ± 14.7	NA	NA
	NR	37.4 ± 26.6 mo	85 ± 26.9	NA	NA	NA	NA
	0%	6.2 ± 0.3 mo	76.8 ± 15	NA	NA	NA	NA
	NR	8.5 ± 4.2 mo	66.7 ± 22.5	NA	NA	NA	NA
	MR ALL = 71.8% NRTSSS = 75% RTSSS = 69.6%	ALL = 8–24 mo^j^ NRTSSS = 11.5 (5.5) mo RTSSS = 12 (5) mo	NRTSSS = 60.8 (34.8) RTSSS = 85 (16.7)	NA	NA	NA	NA
	M ALL = 80% < 56 = 88.2% ≥ 56 = 75.8%	Pre-op (day before surgery)	ALL = 54.8 ± 18 < 56 = 43.2 ± 9.1 ≥ 56 = 60.8 ± 18.9	6 mo^o^	ALL = 63.7 ± 19.2 < 56 = 42 ± 9.4 ≥ 56 = 74.9. ± 11.9	NA	NA
	≥ 50 yr at ACLR M = 68% CL = 76% ≤ 40 years at ACLR M = 38% CL = 10%	Pre-ACLR	Short (6-item) ALL = NR ≥ 50 yr at ACLR = 25 ± 20.2 ≤ 40 yr at ACLR = NR	ALL = NR ≥ 50 yr at ACLR = 14.2, 3.5–30.5 mo ≤ 40 yr at ACLR = 20.5, 11.4–29.4 mo	Short (6-item) ALL = NR ≥ 50 yr at ACLR = 70.8 ± 19.7 ≤ 40 yr at ACLR = 69.1 ± 20.5	NA	NA
	M = 63.2%	25.7 ± 6.2 wk	74.4 ± 25.6	NA	NA	NA	NA
	NR	8.7 ± 5.1 mo	ALL = 64.8 ± 19.5 IRTS = 70.1 ± 15.9 NIRTS = 42.5 ± 17.5	NA	NA	NA	NA
	MM = 14% LM = 34%	6 mo	58.2 / 3.9	12 mo	64.8 / 3.9	24 mo	71.2 / 3.9
	PCL = 0.5% MCL = 4.1% LCL = 2.5% MM = 22.4% LM = 38% Ch = 28.6%	Diagnosis	49.3 ± 26.3 (missing for n = 649)	24 mo	78.7 ± 20.2 (missing for n = 649)	NA	NA
	NR	6 mo	Full (12-Item) NRTS = 51.7 ± 25 RTT = 69.6 ± 19 RTSS = 68.1 ± 20 RTSSS = 81.4 ± 15 Short (6-Item) NRTS = 47.9 ± 26 RTT = 65.6 ± 21 RTSS = 63.7 ± 24 RTSSS = 77.8 ± 18	NA	NA	NA	NA
	NR	12, 8–22 mo	RTS = 70, 11–99 NRTS = 69, 0–92 AG NIRTS = 39.1: 3.2 IRTS = 54.9: 3.3 RTT = 63: 2.4 RTSSS = 76.3: 2.1	NA	NA	NA	NA
	NR	12 mo	ALL = NR RTSSS = 75 ± 21 NRTSSS = 64 ± 21	NA	NA	NA	NA
	NR	12 ± 1 mo	ALL = 65 ± 23 RTSSS = 79 ± 17 NRTSSS = 60 ± 23	NA	NA	NA	NA
	M Surgery ALL = 89.2% SGF = 84% MGF = 95% Non-M Surgeries at 1st R-ACLR ALL = 18.6% SGF = 24% MGF = 11%	SGF = 29 [12–122] mo MGF = 85 [12–272] mo	SGF = 45.1 [0–99.1] MGF = 11 [0–66.7] (missing for n = 60)	NA	NA	NA	NA
	M = 51% Ch = 27.1%	2.8 ± 1.3 yr	Short (6-item) 75.9 ± 23.7	NA	NA	NA	NA
Delayed ACLR without prior exercise therapy	0%	ALL = 35, 12–81 mo RTSS = 34.3 ± 15.1 mo NRTSS = 35.8 ± 15.3	ALL = 4.9 ± 2.1^m^ RTSS = 6.2 ± 2^m^ NRTSS = 4.5 ± 2.1^m^	NA	NA	NA	NA
	M = 50%	1 wk pre-ACLR	ALL = NR RTSSS = 45.2 ± 21.6 NRTSSS = 37.4 ± 18.4	4 mo	ALL = NR RTSSS = 57.3 ± 20.3 NRTSSS = 40.4 ± 17.1	NA	NA
	NR	8 mo	ALL = NR 15–20 yr = 58.3 [17.5–99.2] (missing for n = 58) 21–30 yr = 55 [10–99.2] (missing for n = 61) RMF = 61.3 [17.5–95.8] (n = 44) NRMF = 55 [10 -99.2] (n = 165)	12 mo	ALL = NR 15–20 y = 63.8 [13.3–100] (missing for n = 90) 21–30 y = 58.3 [10–100] (missing for n = 210) RMF = 74.2 [26.7–100] (n = 35) NRMF = 61.3 [13.3 -100] (n = 102)	NA	NA
	MRS = 18% MR = 24% CD = 1% MF = 1%	10.4 mo^h^	ALL = 55.8 ± 22.4 RTSSS = 63.5 ± 20.8 NRTSSS = 50.3 ± 22	NA	NA	NA	NA
	M = 69% Ch = 71%	42.2 mo^h^	73.9 ± 23.3	NA	NA	NA	NA
	O ACLR = 1.7% C ACLR = 6.8% OC = 2.5%	12.7 ± 0.1 mo	53 ± 20	NA	NA	NA	NA
	NR	0.8, 0.1–3.2 mo pre-ACLR	49.2 ± 24.8	3.1, 2.5–4.2 mo	64.5 ± 22.8	6.6, 5–11.2 mo	74.3 ± 19.3
	NR	2–5 yr	ALL = 5 ± 2.2^m^ RTSS = 6.1 ± 1.9^m^ NRTSS = 4.2 ± 2^m^ RTSSS = 7 ± 1.8^m^ NRTSSS = 4.3 ± 1.8^m^ Retest = 5.1 ± 2.1^m^	40 ± 16 d^n^	ALL = NA Retest = 5.2 ± 2.3^l^	NA	NA
	0%	6.2 ± 0.3 mo	48.8 ± 27.3	NA	NA	NA	NA
	M = 64.8% Ch = 22% EAT = 58.4%	Pre-R-ACLR	54.9 ± 23.6 (missing for n = 283)	2 yr	71.5 ± 24.2	NA	NA
	NR	8 mo	ALL = NR LSI ≥ 90% = 51.4^h^^,i^ LSI ≤ 85% = 47.9^h^^,i^ LSI < 85% > 90% = 47.3^h^^,i^	12 mo	ALL = NR LSI ≥ 90% = 70.9^h^^,i^ LSI ≤ 85% = 67.8^h^^,i^ LSI < 85% > 90% = 66.1^h^^,i^	NA	NA
	BPTB = M (11.1%) HT = M (5.6%)	Pre-op R-ACLR	BPTB = 55.3 ± 15.2 (missing for n = 25) HT = 49.4 ± 21.4 (missing for n = 21)	BPTB = 56 mo HT = 57 mo	BPTB = 74.8 ± 21 HT = 70.9 ± 26.3	NA	NA
	MCL = 4.5% LCL = 2.4% MM = 31% LM = 38.7% Ch = 38.7%	Diagnosis	40.3 ± 26 (missing for n = 132)	24 mo	41.8 ± 25.6 (missing for n = 132)	NA	NA
Delayed ACLR	ALL = LM (28%), MM (14%) Auto = LM (20%), MM (8%) Allo = LM (32%), MM (20%)	60 mo	ALL = 83 ± 24.9 Auto = 78.8 ± 23.7 Allo = 87.5 ± 25.9	NA	NA	NA	NA
	M = 66% Ch = 50%	49.6 ± 24 mo	76.2 ± 32.2	NA	NA	NA	NA
Rehabilitation Only	NR	“Pre-op”	30.2 ± 26.1	18 mo^n^	60.7 ± 27.2	NA	NA
	0%	> 12 months post-injury^n^	68.3 ± 17.8 (missing for n = 8)	NA	NA	NA	NA
Unclear	NR	11.2 ± 3.8 mo	Short (6-item) ALL = 52.3 ± 21.6 RTS = 59.9 ± 19.6 NRTS = 42.3 ± 20.2	NA	NA	NA	NA
	MM = 42% LM = 37% Ch = 44% HG-Ch = 8%	12 mo	55.7 ± 23.9	NA	NA	NA	NA
	NR	6 mo	ACLR = 67.7 ± 17.2 Retest = 62 ± 14.2	8 mo	ACLR = NR Retest = 79.4 ± 13.6	NA	NA
	NR	6 mo	All = 62.8 ± 19.4 RTSS = 72.1 ± 21.4 NRTSS = 60.3 ± 18.1	NA	NA	NA	NA
	M = 40%	19 ± 4.6 mo	ALL = 95.6 ± 6 R = 97.6 ± 5.3 CP = 100 ± 10.1	NA	NA	NA	NA
	M^r^ ALL = 50.5% C = 0% MR = 100% MS = 100%	C = 43.15 ± 2.3 wk post surgery Meniscectomy: 42.65 ± 2.3 wk post surgery Repair: 42.28 ± 2.6 wk post surgery	C = 75.4 (24.2) MS = 80 (25) MR = 82.5 (19.4)	NA	NA	NA	NA
	NR	15.6 ± 1.9 mo	61.3 ± 17.8	NA	NA	NA	NA
	NR	18 mo^i^	ALL = NR RTSS = 6.5 ± 1.9^l^ NRTSS = 3.8 ± 1.9^l^	NA	NA	NA	NA
	NR	6.5 ± 1 mo	67.8^h^	NA	NA	NA	NA
	NR	ALL = 9 ± 2.3 mo S = 9.1 ± 2.3 mo L = 8.9 ± 2.6 mo	ALL = NR S = 68 ± 18.5 L = 65.3 ± 20	NA	NA	NA	NA
	NR	13.2 mo^h^	ALL = NR < 7 pts (Likert) = 60.3 ± 27 ≥ 7 pts (Likert) = 72.2 ± 22.1	NA	NA	NA	NA
	0%	6 mo	52.7 ± 22.5	NA	NA	NA	NA
	NR	13.6 ± 11 mo	53.5 ± 21.6	NA	NA	NA	NA
	NR	8.2 ± 6.9 mo	60.1 ± 19.2	NA	NA	NA	NA
	NR	≥ 6 mo	ALL = 56.2 ± 16.5 RTSSS = 65.1 ± 14.3 NRTSSS = 51 ± 15	NA	NA	NA	NA
	ALL = MM (21.2%), LM (22.4%) RTS = MM (16.4%), LM (20.9%) NRTS = MM (38.9%), LM (27.8%)	6 mo	ALL = 42.6 ± 16.5 RTS = 43.5 ± 18.6 NRTS = 31.3 ± 13.6	12 mo	ALL = 50.8 ± 19.9 RTS = 54.1 ± 20.7 NRTS = 31.4 ± 13.3	24 mo	ALL = 58.6 ± 24.3 RTS = 62.3 ± 22.7 NRTS = 34.3 ± 16.6
	NR	6.8, 5–9 mo	ALL = NR F = 75 [5.8–98.3] M = 82.5 [35–100]	NA	NA	NA	NA
	ALL = 67.3% A = 75.5% B = 59.2%	9 mo	ALL = NR A = 53.6 ± 6.4 B = 57 ± 5.8	24 mo	ALL = NR A = 63.7 ± 8.1 B = 67.5 ± 7.2	NA	NA
	NR	ALL = 6 mo^h^ Int = 6.1, 3.5–8 mo C = 6.5, 4.5–9.5 mo	ALL = NR C = 66.7 ± 22.2 Int = 62.1 ± 18.2	ALL = 1–2 wk^n^ C = pre-intervention Int = post-intervention	ALL = NR C = 71.1 ± 20.5 Int = 58.7 ± 15.8	NA	NA
	NR	2 wk pre-ACLR^h^	Short (6-item) ALL = 49.5 ± 21.8 ≤ 20 yr = 51.9 ± 21.2 > 20 yr = 47.9 ± 22.1 Inj = 53.4 ± 24.5 NInj = 48.8 ± 21.1	12 mo	Short (6-item) ALL = 66.4 ± 22.4 (missing for n = 47) Inj = 60.9 ± 23.4 NInj = 67.2 ± 22.2 ≤ 20 yr = 68.7 ± 20.5 > 20 yr = 64.1 ± 23.9	NA	NA
	NR	9 mo	74.17 (59.2–86)	NA	NA	NA	NA
	MR = 80.4%	16 wk	ALL = NR ISI = 55.3 ± 12.4 OSI = 60.8 ± 11.6	NA	NA	NA	NA
	NR	Pre-ACLR	Int = 5.2 ± 1.2^l^ P = 5.1 ± 2.3^l^ C = 6.1 ± 1.6^l^	Hospitalization	Int = 5.6 ± 1^l^ P = 5.5 ± 2^l^ C = 6.2 ± 1.3^l^	2 wk	Int = 5.7 ± 1.2^l^ P = 5.6 ± 2^l^ C = 5.8 ± 1.4^l^^,p^
	40.3%	44.1 ± 17.8 mo	67.3 ± 24.8	NA	NA	NA	NA
	EAT = 28.6% C = 23.3% MM = 37.2% LM = 32%	Pre-ACLR	41.3 ± 25.4	4 mo	55.1 ± 21.3	6 mo	ALL = 58.3 ± 22.3 RTSS = 70.6 ± 19.4 NRTSS = 55.3 ± 22^q^
	NR	6 mo = 6 mo 12 mo = 12 mo	ALL = 69.9 ± 20.8 6 mo = 68.1 ± 21 12 mo = 73.4 ± 20.3 RTSSS = 80 ± 12.6 NRTSSS = 50.2 ± 19.6	NA	NA	NA	NA
	NR	ALL = NR LSI > 90% = 8.9 ± 3 mo LSI ≤ 90% = 8.4 ± 2.5 mo	ALL = NR LSI > 90% = 85 (68.1) LSI ≤ 90% = 70 (46.7)	NA	NA	NA	NA
	NR	9.5 ± 4 mo	56.5 ± 22.2	NA	NA	NA	NA
	NR	5 ± 1.6 mo	55.8 ± 21.6	80 ± 19.3 d^n^	60.1 ± 22.5	NA	NA
	NR	6.4 ± 2.1 mo	65.8 (57.5–79.2)	NA	NA	NA	NA
	NR	9 ± 5 mo	62.6 ± 20.9	NA	NA	NA	NA
	MM = 26.8% LM = 34.9% Ch = 20%	6.3 ± 0.5 mo	Full Form ALL = 54.4 ± 22 F = 52.3 ± 21.8 M = 55.8 ± 22.1 Short Form ALL = 49.4 ± 22.7 F = 47.3 ± 22.9 M = 50.9 ± 22.4	12 ± 0.5 mo	Full Form ALL = 66.8 ± 23.7 F = 64.2 ± 23.5 M = 68.7 ± 23.7 Short Form ALL = 63.3 ± 24.6 F = 60.4 ± 24 M = 65.3 ± 24.9	NA	NA
	NR	6.5 ± 0.7 mo	61.7 ± 16.6	9.5 ± 0.6 mo	67.3 ± 18.1	NA	NA
	NR	19, 6–57 mo	89.1 ± 12.9	NA	NA	NA	NA
	54.2%	30.7 ± 18.9 mo post-R-ACLR	52.1 ± 27.1	NA	NA	NA	NA
	NR	ALL = NR Low = 24.1 ± 8.8 wk Mid = 23.3 ± 7.1 wk High = 23.3 ± 9.2 wk	ALL = 61^i^ Low = 34 ± 11 Mid = 62 ± 9 High = 90 ± 6	NA	NA	NA	NA
	NR	ALL = NR Re = 24.6 ± 8.1 wk Non = 22 ± 7.4 wk	ALL = NR Re = 56.7 ± 19.9 Non = 59.9 ± 13.2	ALL = NR Re = 31.9 ± 8.2 wk Non = 29.3 ± 7.8 wk	ALL = NR Resp = 79 ± 17.7 Nonresp = 58.4 ± 13.4	NA	NA

^a^ACL Injury Treatment Categories: Surgical reconstruction occurred within 6 months of ACL injury without attempting exercise therapy or following a period of “pre-habilitation” with the intention of undergoing surgery on completion. (Early ACLR), Surgical reconstruction occurred after 6 months of ACL injury without attempting exercise therapy or following a period of “pre-habilitation” with the intention of undergoing surgery on completion. (Delayed ACLR w/o prior exercise therapy) Surgical reconstruction that occurred following a trial of management with rehabilitation alone (i.e. exercise therapy). Patients may have ‘crossed-over’ to ACL surgery for a number of reasons including episodes of functional knee instability, patient choice, surgeon recommendations, inability to meet strength/function milestones. (Delayed ACLR), Surgical repair of the native ACL ligament with or without rehabilitation prior to surgery. (ACL-Repair), Individuals who commenced a standardized or criterion-based rehabilitation program within 6 months of injury and did not undergo surgical intervention for their ACL injury. (Rehabilitation only)

^b^Study Design: Case Control (CC), Cross-Sectional (C-S), Case Series (CS), Prospective Cohort (PC), Retrospective Cohort (RC), Randomized Controlled Trial (RCT), Intervention Study (non-randomized) (IS)

^c^Standardized Pre-injury Activity Level: Recreational = Tegner ≤ 6, inactive individuals, < 4 h/wk or < 4 d/wk of activity, L3 on pivoting/cutting scales (Rec), Competitive = Tegner ≥ 7, > 4 h/wk and/or > 4 d/wk of activity, L1 or L2 on pivoting/cutting scales (Comp) Elite = Tegner 10, collegiate/professional athletes, ^Unspecified^ = no information present (U)

^d^Intent to RTS: Yes = 'intent to RTS' in selection criteria, they say people attempted RTS or if they report a RTS%, it is higher than 50% (Y), No = explicitly say something like 'people were counselled to not RTS' or 'To change activity level' or something to that effect or RTS% is less than 50% (N), Unspecified = no information present (U)

^e^Injury Classification: Meniscal (M), Medial Meniscal (MM), Lateral Meniscal (LM), Chondral (Ch), High-Grade Chondral (HG-Ch), Extra-articular Tenodesis (EAT), Cartilage Lesions (CL), Medial Collateral Ligament (MCL), Posterior Cruciate Ligament (PCL), Lateral Collateral Ligament (LCL), Meniscal Repair (MR), Meniscal Resection (MRS), Meniscectomy (MS), Cartilage Debridement (CD), Microfracture (MF), Patellofemoral Cartilage Lesion (PFCL), Other injury/surgery on the ACLR Limb (O ACLR), Contralateral limb ACLR injury/surgery (C ACLR), Other contralateral limb injuries/surgeries (OC)

^f^Post-ACL treatment

^g^Median

^h^Age at baseline

^i^Reported by author

^j^Mean

^k^Age collection time not specified

^l^Range

^m^0-10 ACLRSI Score

^n^Post-Baseline

^o^Final follow-up or, before the 2nd injury with progression to a complete ACL tear

^p^TP4 for ACL-RSI^m^: 6 wk; TP4 ACL-RSI Results: Int = 5.9 ± 0.8, P = 6.1 ± 1.6, C = 6.2 ± 1.3; TP5 for ACL-RSI: 3 mo; TP5 ACL-RSI Results: Int = 5.3 ± 1.2, P = 5.8 ± 1.1^l^, C = 6 ± 1.3, TP6 for ACL-RSI: 6 mo; TP6 ACL-RSI Results: Int = 5.9 ± 1.4, P = 6.3 ± 1.8, C = 6.6 ± 1.2

^q^TP4 for ACL-RSI: 1 yr; TP4 ACL-RSI Results: ALL = 64.7 ± 24.2, RTSS = 74.1 ± 19.8, NRTSS = 53.8 ± 24.3; TP5 for ACL-RSI: 2 yr; TP5 ACL-RSI Results: ALL = 65.2 ± 25.3, RTSS = 75.7 ± 19.3, NRTSS = 50.6 ± 25.6, RTSSS = 81.6 ± 16.1, NRTSSS = 53.2 ± 24.1

^r^does not include tears deemed stable by surgeon

^s^Number of knees, participants (n = 174)

ACLR = Anterior Cruciate Ligament Reconstruction; R-ACLR = Revision Anterior Cruciate Ligament Reconstruction

RTS Classification: Return to Sport at Any Level (RTS), Return to Same Sport at Any Level (RTSS), Return to Same Sport at Same or Higher Level (RTSSS), Return to Training (RTT)

TP1 ACL-RSI (TP1), TP2 ACL-RSI (TP2), TP3 ACL-RSI (TP3), TP4 ACL-RSI (TP4), TP5 ACL-RSI (TP5), TP6 ACL-RSI (TP6)

Group Classification: Intervention (Int), Control (C), Placebo (P), In-Sport Injury (ISI), Out-of-Sport Injury (OSI), Intention to Return to Sport (IRTS), No Intention to Return to Sport (NIRTS), High Perceived Disability (HPD), Low Perceived Disability (LPD), Male (M), Female (F), Recovered Muscle Function (RMF), Not Recovered Muscle Function (NRMF), Limb Symmetry Index (LSI), 2nd ACL Injury After RTS Post-1st ACLR (Inj), No 2nd ACL Injury After RTS Post-1st ACLR (NInj), Quadricep Graft (QT), Hamstring Graft (HT), Single Graft Failure (SGF), Multiple Graft Failure (MGF), Patellofemoral pain (PFP), No Patellofemoral pain (NPFP), ACL-RSI ≤ 47 (Low), 48 ≤ ACL-RSI ≤ 78 (Mid), ACL-RSI score ≥ 79 (High), tunnel widening was present, either tunnel ≥ 12 mm (≥ 12 mm), tunnel widening not present, both tunnels < 12 mm (< 12 mm), Subgroup that was retested (Retest), increase in the ACL-RSI score ≥ 10 points (Re), change of

< 10 points (Non), ACL-RSI ≥ 56 (≥ 56), ACL-RSI < 56 (< 56), height ≤ 163 cm (S), height > 163 cm (L), four single leg HOP test results scored above 90% of limb symmetry index (LSI > 90%), at least one single leg HOP test result scored lower than 90% of LSI (LSI ≤ 90%), ACL + anterolateral ligament reconstruction using hamstring tendon graft (HT-ALL), bone-patellar tendon-bone graft + modified Lemaire tenodesis procedure (BPTB-L), age 16–20 year (A), age 21–45 year (B), Meniscal Repair (MR), Meniscectomy (MS), recreational according to self-reported Return to Play Level based on the TALS post-injury (R), competitive according to self-reported Return to Play Level based on the TALS post-injury (CP), hamstring autograft (Auto), allograft tendon (Allo)

Mean ± SD

Mean: SE

Mean / SEM

Mean, Range

Median (IQR)

Median [Range]

Day (d)

Week (wk)

Month (mo)

Year (yr)

Additional Grouping (AG)

Mean/median participant age at the time of ACL treatment ranged from 15 to 61 years [71, 105]. The proportion of female participants ranged from 0 to 100% [55, 62, 89]. The proportion of participants with concomitant injuries ranged from 0 to 89% amongst included studies [62, 83, 85, 112]. The majority of studies included participants who were active in sport, either in preinjury competitive sport (38 original studies, 46%),[13, 15, 21, 38, 39, 40, 41, 42, 45, 50, 54, 57, 58, 59, 63, 64, 67, 69, 70, 72, 73, 76–78, 80, 83, 85, 86, 89, 91, 92, 96, 97, 100, 102, 108, 111, 113, 114], or recreational sport (10 original studies, 12%) [43, 44, 53, 68, 74, 82, 103, 107, 109]. Only one subgroup of participants in one study was classified as elite athletes [77].

### Risk of Bias and Evidence Synthesis

Sixty-five studies were judged to be at high risk of bias (78%) [13, 15, 16, 19, 21, 37, 41, 43, 45, 46, 48, 50–54, 56–62, 65–71, 73–78, 80, 81, 83–95, 96–100, 103–106, 111–115]. Thirty-eight (46%) studies had concerns for high or serious risk of bias for confounding [19, 41, 42, 45, 48, 52, 53, 59, 62, 65, 68, 71, 73–75, 77, 78, 81, 83, 84, 86–88, 90, 91, 93, 96, 97, 102–105, 108, 111–115], 54 (65%) studies were judged to be at high or unclear risk for missing data [13, 15, 16, 19, 40, 41, 43, 45, 48, 50–54, 56–59, 61, 62, 64, 66–68, 70, 73–77, 80, 81, 83–85, 87–95, 96, 97, 99, 101, 104–106, 111–113], and 37 (45%) studies were judged to be at high or unclear risk for selection bias [13, 15, 16, 19, 21, 37, 43, 45–49, 54, 56, 59–63, 65, 67, 71, 73, 75, 80, 84, 86, 88, 95, 97–99, 106, 107, 110, 114, 115]. Risk of bias judgements are provided in “Additioanl file 1: Appendix 3”. Based on the risk of bias judgements and the number of studies per meta-analysis, the strength of evidence was judged to be weak for the pooled ACL-RSI scores from all studies (at all time-points), early-ACLR (at all time-points except the 1 to 2 year time-point where there was no evidence), delayed ACLR (pre-ACLR and 3 to 6 months post-ACLR time points, all others are limited evidence), unclear treatment (at all time-points except 1 to 2 years post-ACLR where there was no evidence), and for the sensitivity analysis (at all time-points except for the 1 to 2 years post-ACLR time-point where there was no evidence).

### Pooled ACL-RSI Scores

**Fig. 2:**
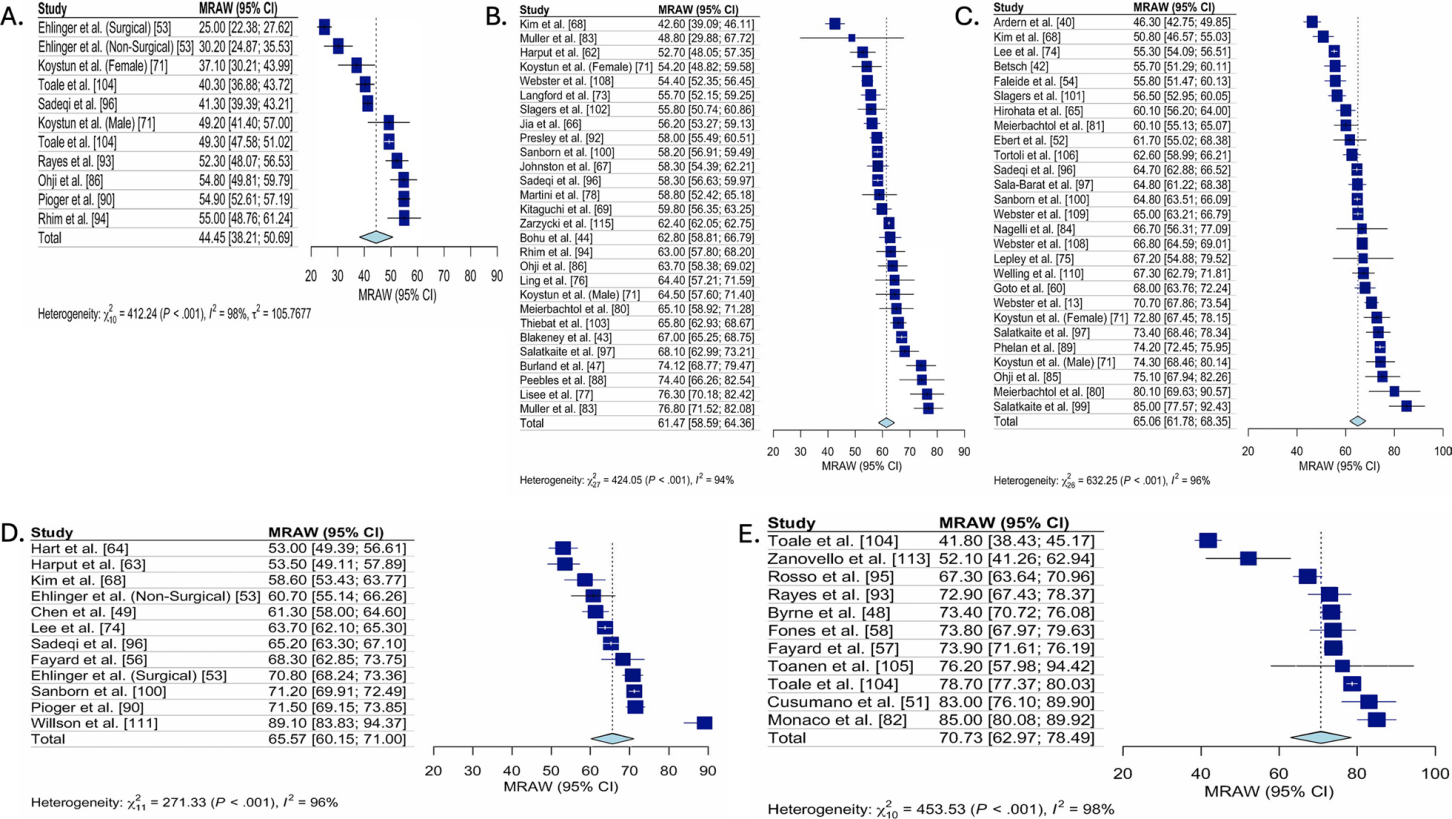
Forest Plots for the pooled ACL-RSI scores for all studies, stratified by time. Forest plot for the pooled mean (95%Confidence Interval [CI]) ACL-RSI score for all studies, stratified by time since ACL tear. **A** Forest plot for ACL-RSI scores pooled mean (95% confidence interval) pre-ACLR. **B** Forest plot for ACL-RSI scores pooled mean (95% confidence interval) from 3 to 6-months post ACL tear. **C** Forest plot for ACL-RSI scores pooled mean (95% confidence interval) from 7 to 12-months post ACL tear. **D** Forest plot for ACL-RSI scores pooled mean (95% confidence interval) from 1 to 2-years post ACL tear. **E** Forest plot for ACL-RSI scores pooled mean (95% confidence interval) from 2 to 5-years post ACL tear. ACL-RSI, Anterior Cruciate Ligament Return to Sport Index; MRAW, Raw mean was used for analysis

**Fig. 3:**
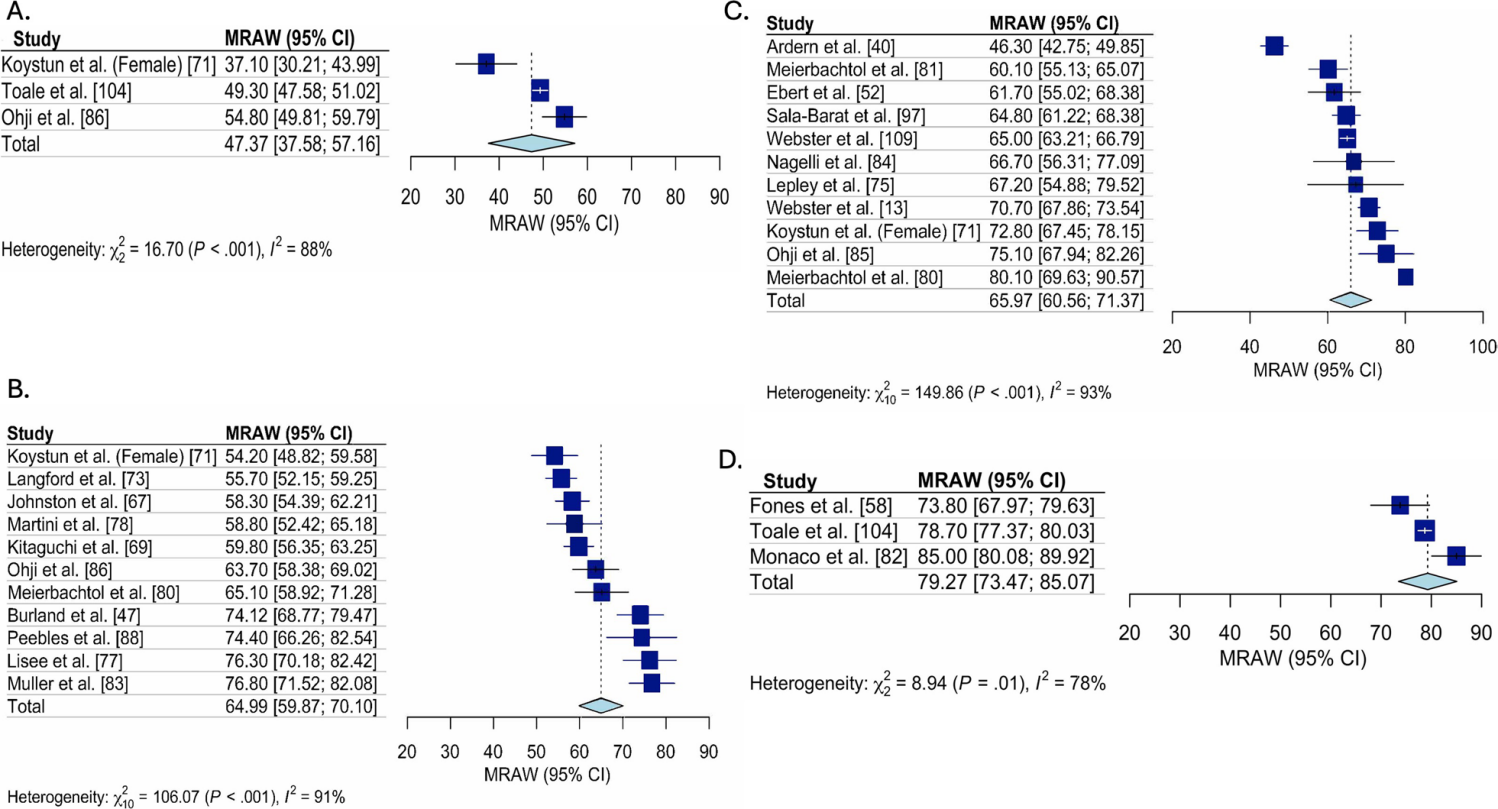
Pooled ACL-RSI scores for only early ACLR studies, stratified by time. Forest plot for the pooled mean (95%Confidence Interval [CI]) ACL-RSI score for all studies classified as early ACLR, stratified by time since ACL tear. **A** Forest plot for ACL-RSI scores pooled mean (95% confidence interval) pre-ACLR. **B** Forest plot for ACL-RSI scores pooled mean (95% confidence interval) from 3 to 6-months post early ACLR. **C** Forest plot for ACL-RSI scores pooled mean (95% confidence interval) from 7 to 12-months post early ACLR. **D** Forest plot for ACL-RSI scores pooled mean (95% confidence interval) from 2 to 5-years post early ACLR. ACL-RSI, Anterior Cruciate Ligament Return to Sport Index; MRAW, Raw mean was used for analysis

**Fig. 4:**
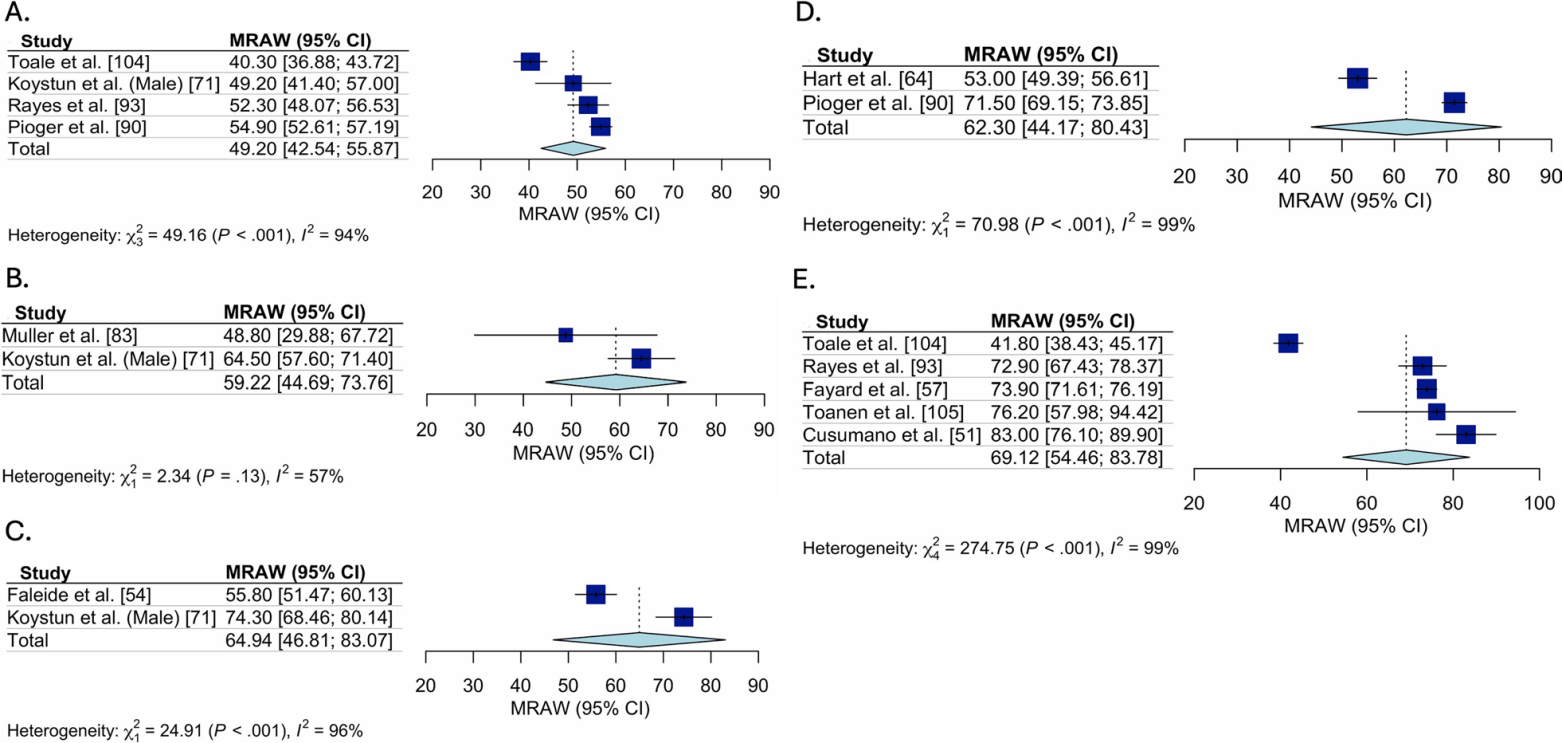
Pooled ACL-RSI scores for only late ACLR studies, stratified by time. Forest plot for the pooled mean (95% Confidence interval [CI]) ACL-RSI score for all studies classified as delayed ACLR, stratified by time since ACL tear. **A** Forest plot for ACL-RSI scores pooled mean (95% confidence interval) pre-ACLR. **B** Forest plot for ACL-RSI scores pooled mean (95% confidence interval) from 3 to 6-months post late ACLR. **C** Forest plot for ACL-RSI scores pooled mean (95% confidence interval) from 7 to 12-months post late ACLR. **D** Forest plot for ACL-RSI scores pooled mean (95% confidence interval) from 1 to 2-years post late ACLR. **E** Forest plot for ACL-RSI scores pooled mean (95% confidence interval) from 2 to 5-years post late ACLR. ACL-RSI, Anterior Cruciate Ligament Return to Sport Index; MRAW, Raw mean was used for analysis

**Fig. 5:**
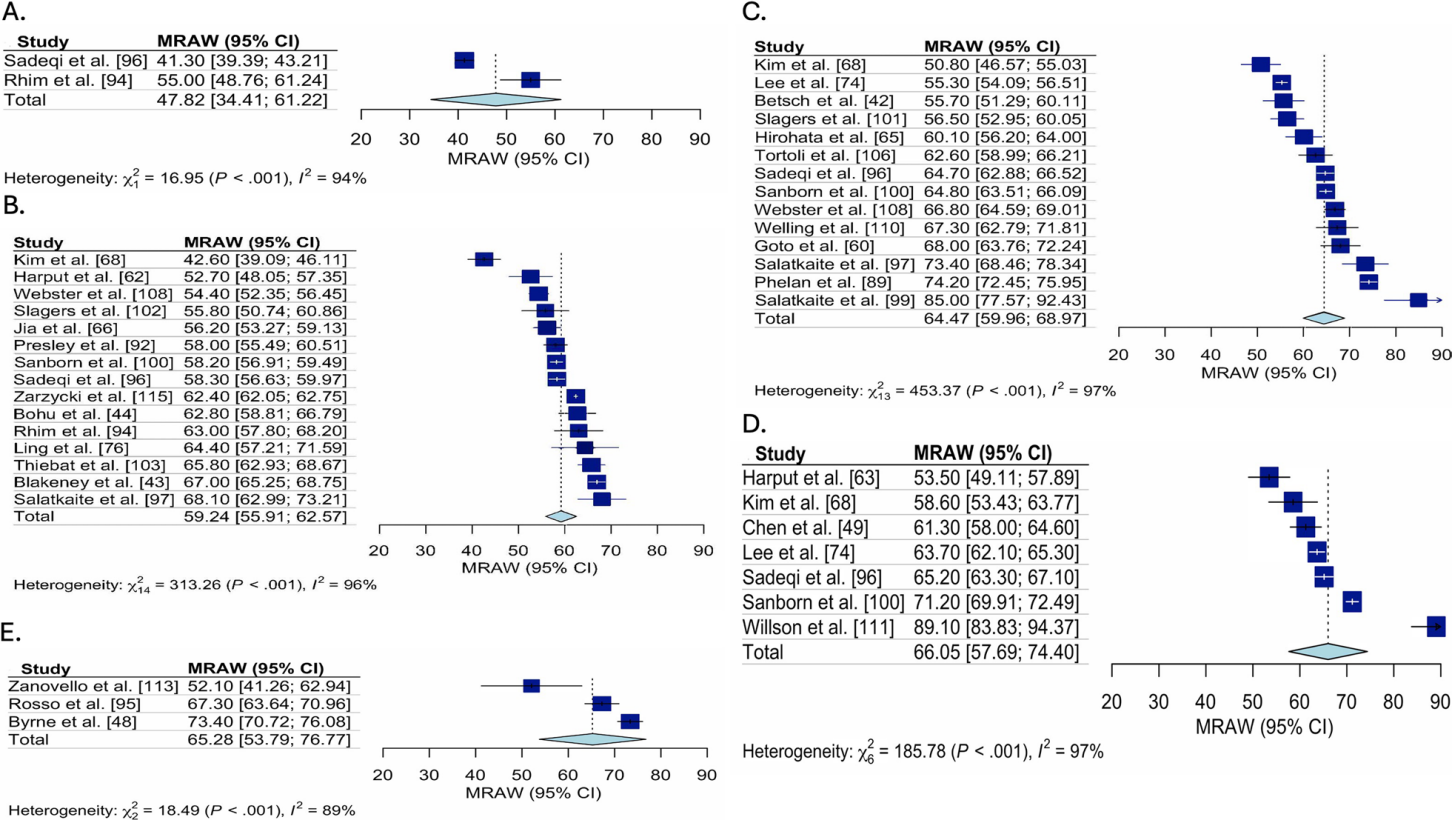
Pooled ACL-RSI scores for all unclear studies, stratified by time. Forest plot for the pooled mean (95%Confidence Interval [CI]) ACL-RSI score for all studies with unclear treatment strategy, stratified by time since ACL tear. **A** Forest plot for ACL-RSI scores pooled mean (95% confidence interval) pre-ACLR. **B** Forest plot for ACL-RSI scores pooled mean (95% confidence interval) from 3 to 6-months post ACL tear. **C** Forest plot for ACL-RSI scores pooled mean (95% confidence interval) from 7 to 12-months post ACL tear. **D** Forest plot for ACL-RSI scores pooled mean (95% confidence interval) from 1 to 2-years post ACL tear. **E** Forest plot for ACL-RSI scores pooled mean (95% confidence interval) from 2 to 5-years post ACL tear. ACL-RSI, Anterior Cruciate Ligament Return to Sport Index; MRAW, Raw mean was used for analysis

**Fig. 6:**
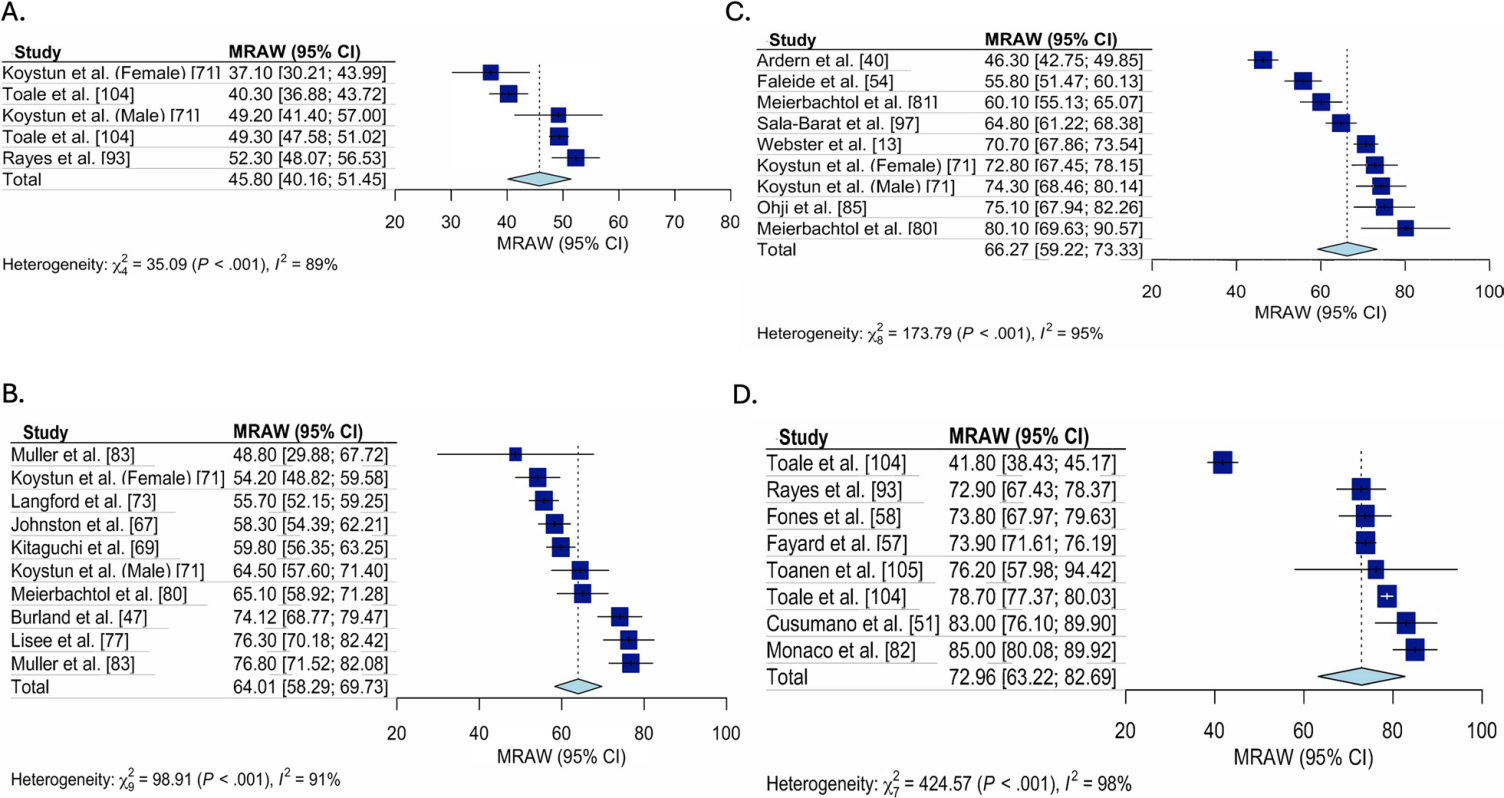
Pooled ACL-RSI scores for studies where there was an intention to RTS, stratified by time. Forest plot for the pooled mean (95%Confidence Interval) ACL-RSI score for all studies that were judged to have an intent to return-to-sport, stratified by time since ACL tear. **A** Forest plot for ACL-RSI scores pooled mean (95% confidence interval) pre-ACLR. **B** Forest plot for ACL-RSI scores pooled mean (95% confidence interval) from 3 to 6-months post ACL tear. **C** Forest plot for ACL-RSI scores pooled mean (95% confidence interval) from 7 to 12-months post ACL tear. **D** Forest plot for ACL-RSI scores pooled mean (95% confidence interval) from 2 to 5-years post ACL tear. ACL-RSI, Anterior Cruciate Ligament Return to Sport Index; MRAW, Raw mean was used for analysis

Meta-analysis results pooling ACL-RSI scores for all studies, for studies categorized as early ACLR, delayed ACLR and unclear, and stratified by time point (pre-ACLR, 3 to 6 months post-ACLR, 7 to 12 months post-ACLR, 1 to 2 years post-ACLR, and 2 to 5 years post-ACLR) are summarized in Table 2. The Forest plots for each meta-analysis performed are presented in Figs. 2, 3, 4, 5 and 6 and represent the pooled ACL-RSI scores (each stratified by time point) for all studies (Fig. 2), pooled ACL-RSI scores for early ACLR studies (Fig. 3), pooled ACL-RSI scores for late ACLR studies (Fig. 4), pooled ACL-RSI scores for all unclear studies (Fig. 5), and pooled ACL-RSI scores for studies where there was an intention to RTS (Fig. 6).

**Table 2:** Pooled ACL-RSI scores based on treatment strategy, stratified by follow up time

Treatment Category	Time period				
	Pre-ACLR	3–6 months post-ACLR	7–12 months post-ACLR	1–2 years post-ACLR	2–5 years post-ACLR
All studies	44.4 (38.2, 50.7, I^2^ = 98%, n = 11)	61.5 (58.6, 64.4, I^2^ = 94%, n = 28)	65.1 (61.8, 68.4, I^2^ = 96%, n = 27)	65.6 (60.1, 71.0, I^2^ = 96%, n = 12)	70.7 (63.0, 78.5, I^2^ = 98%, n = 11)
Early ACLR	47.4 (37.6, 57.2, I^2^ = 76%, n = 3)	65.0 (59.9, 70.1, I^2^ = 91%, n = 11)	66.0 (60.6, 71.4, I^2^ = 93%, n = 11)	Unable to calculate	79.3 (73.5, 85.1, I^2^ = 78%, n = 3)
Late ACLR	49.2 (42.5, 55.9, I^2^ = 94%, n = 4)	59.2 (44.7, 73.8, I^2^ = 68%, n = 2)	64.9 (46.8, 83.1, I^2^ = 96%, n = 2)	62.3 (44.2, 80.4, I^2^ = 99%, n = 2)	69.1 (54.5, 83.8, I^2^ = 99%, n = 5)
Unclear	47.8 (34.4, 61.2, I^2^ = 94%, n = 2)	59.2 (55.9, 62.6, I^2^ = 96%, n = 13)	64.5 (60.0, 69.0, I2 = 97%, n = 14)	66.1 (57.7, 74.4, I^2^ = 97%, n = 7)	65.3 (53.8, 76.8, I^2^ = 98%, n = 3)
Intention to RTS = yes	45.8 (40.2, 51.5, I^2^ = 88%, n = 5)	64.0 (58.3, 69.7, I^2^ = 91%, n = 10)	66.9 (60.2, 73.7, I^2^ = 95%, n = 9)	Unable to calculate	73.0 (63.2, 82.7, I^2^ = 98%, n = 8)

Results are pooled mean (95%Confidence Interval, I^2^, number of studies), ACL-RSI (anterior cruciate ligament return-to-sport after injury scale), ACLR (anterior cruciate ligament reconstruction), RTS (return-to-sport)

ACL-RSI scores were higher 3 to 6 months post ACLR (pooled mean = 61.5 [95% CI 58.6, 64.4], I^2^ = 94%) compared to pre-ACLR (pooled mean = 44.4 [95% CI 38.2, 50.7], I^2^ = 98%), then remained stable at 6 to 12 month post ACLR (pooled mean = 65.1 [95% CI 61.8, 68.4], I^2^ = 96%) and 1–2 years post ACLR (pooled mean = 65.6 [95% CI 60.1, 71.0], I^2^ = 96%), before being highest 2 to 5 years post ACLR (pooled mean = 70.7 [95% CI 63.0, 78.5], I^2^ = 98%). These trends were consistent across treatment approaches (early, delayed and unclear). Two to 5 years post ACLR, early ACLR had higher ACL-RSI scores than delayed (pooled mean = 79.3 [95% CI 73.5, 85.1], I^2^ = 78% vs. pooled mean = 69.1 [95% CI 54.5, 83.8], I^2^ = 99%). Funnel plot assessment revealed no concerns for publication bias (“Additioanl file 1: Appendix 4”).

### Meta-Regression

Due to the paucity of data in different time periods, meta-regressions were only performed for the 3 to 6 months post-ACLR and 1 to 2 years post-ACLR time periods. The results of these two meta-regression analyses (full model) including age, percent female, and time from injury to treatment are presented in “Additioanl file 1: Appendix 5” a, b. These factors explained 47.6% of variance in ACL-RSI scores measured between 3 to 6 months after ACLR (n = 9 studies). For ACL-RSI scores measured between 1 and 2 years after ACLR, these factors explained 27.0% of variance (n = 11 studies).

### Sensitivity Analyses

The sensitivity analysis results restricting the primary meta-analysis to only studies where we judged there was an intention for participants to RTS are reported in Table 1. There were no notable differences at each respective time-point compared to the primary meta-analysis as all time points were considered to be similar to the primary analyses.

## Discussion

There is weak evidence that ACL-RSI scores, regardless of treatment strategy (early versus late ACLR), appear to follow a similar trajectory. The lowest values were seen after injury and prior to surgery (~ 44–49/100), with an increase 3 to 6 months post-surgery (~ 59–65/100), followed by relatively no improvement from 6 to 24-months after ACLR (~ 62–65/100). ACL-RSI scores were highest 2 to 5 years after surgery, regardless of strategy (~ 65 to 79/100). However, those who were classified as early ACLR (i.e., ACLR within a mean time of 6 months from ACL injury) had the highest ACL-RSI scores (mean = 79.3 [95% CI 73.5, 85.1], I^2^ = 78%) 2 to 5 years after ACLR. Despite improvements in ACL-RSI scores (from ~ 40/100 (pre-ACLR) to ~ 70/100 (2–5 years post-ACLR), it is clear that impairments in psychological readiness persist for many individuals beyond 2 years. Further, there is a need for more high quality evidence assessing psychological factors after ACL injury because over 70% of studies were judged to be at high risk of bias [34].

### ACL-RSI Scores Over Time

The pooled ACL-RSI scores across all studies were higher at 3 to 6 months post-ACLR (pooled mean = 61.5 [95% CI 58.6, 64.4])) compared to pre-ACLR (pooled mean = 44.4 [95% CI 38.2, 50.7])). These findings were similar to the concurrent review by our group, which found that TSK-17, TSK-11, and Knee Self-Efficacy Scale improved from the pre-operative time period to the 7 to 12 month time period, but then levelled off at 7 to 12 months following surgery [34]. According to Slager et al.[102] and Webster & Feller [108], the ACL-RSI standard error of measurement (SEM) ranges from 5.5–9.6 and individual minimally important change ranges from 13.4–15 points. Both of these are lower than the differences observed between pre-ACL and all other time points in the current study. However, from 3 to 6, 6 to 12 and 12 to 24 month time points, the differences between each are lower than established SEMs. This fact suggests that these values are identical from a clinical perspective. Application of the minimally important change provides clinical context but may be limited in direct interpretation because this review did not track longitudinal change in individual studies (i.e., within cohort change) and collated different studies (with different participants) at different time points.

The pooled results of this study raise concerns that many individuals are cleared for RTS between 6 to 12 months[116] and there is very little improvement in psychological readiness, measured via ACL-RSI, after the 3 to 6 months post-ACLR. Individuals after ACLR who experience a smaller improvement in ACL-RSI scores over the course of rehabilitation could be at risk for a second ACL injury [79]. Further, only one of the pooled results (early ACLR) had scores better than a previously published cut-off score (76.7/100, 78% sensitive, 39% specific) that identified individuals who went on to sustain a second ACL injury [19]. These are concerning findings and support that, on average, athletes are returning with some important deficits related to fear or confidence. Considering the ACL-RSI is unrelated to other important impairments and common clinical intervention targets (i.e., strength, and function), there is a need for specific interventions targeting psychological factors [117]. Further, a previous systematic review indicated that athletes who returned to sport had higher ACL-RSI scores than those who did not [118]. This review reported nearly identical pooled ACL-RSI scores (pooled mean = 70.9 [95% CI 65.3, 77.0]) to the current review, for those who had already returned to sport compared to the current study [118]. The current review did not seek to stratify those who did and did not RTS, but rather sought to assess factors prior to RTS that may assist clinicians in identifying patients who may struggle to improve psychological health after ACL injury.

### ACL-RSI Scores Based on Treatment Strategy

Although we were unable to directly compare treatment strategies, we performed stratified meta-analyses to provide descriptive data on how ACL-RSI scores may vary based on surgical timing. We are unaware of any previous reviews collating these data. Regardless of treatment strategy, pre-ACLR ACL-RSI scores were similar. This is intuitive as all participants (no matter the treatment strategy) are unlikely to be remotely prepared for RTS and are focused more on the immediate impairments (i.e., swelling, range of motion, muscle activation). While scores were slightly higher for the early vs. delayed group 3–6 months after ACLR, these values were within measurement error (see above) [108]. Further, it is likely that someone who delayed ACLR may feel RTS is a bigger challenge because these athletes were unable to reach their goals through their initial treatment choice and now have a longer recovery ahead. However, by the time RTS typically occurs (7 to 12-months post ACLR), both early and delayed ACLR groups had essentially the same pooled ACL-RSI scores (early ACLR: pooled mean = 66.0 vs. delayed ACLR: pooled mean = 64.9). Considering there appear to be no major differences in other relevant outcomes (kinesiophobia, knee self-efficacy, and fear avoidance) between early vs. delayed ACLR [34, 119–121], the current results provide preliminary descriptive support that these treatment decisions should be contextual and individualized.

We performed a sensitivity analysis to account for the inherent selection bias when assessing RTS outcomes and treatment approach (early ACLR vs. delayed ACLR). Specifically, individuals who are managed non-operatively/delayed ACLR are instructed to avoid RTS at their prior level. Each meta-analysis was repeated including only studies for which we felt there was an intention to RTS. While this is difficult to know, we used previously published RTS prevalence data to inform this decision. Specifically, if RTS prevalence was markedly higher than that reported in the literature, further inspection of selection bias was performed [28, 29]. The results of this sensitivity analysis suggest there was no evidence for a selection bias in our results. Specifically, ACL-RSI scores were consistent with the primary meta-analysis at all time points (Table 2). We acknowledge that the definition of ‘intention’ could have misclassified individuals. This could have lowered the pooled estimates [118].

### Time from Injury to Surgery, Sex, and Age and the Association with ACL-RSI Scores

Understanding non-modifiable (and modifiable) factors related to ACL-RSI scores can help clinicians forecast who may need additional treatment [20]. It is well established that females[22] and younger individuals[5] typically have worse outcomes after ACLR. The results of our meta-regression suggest that these factors are also important in psychological readiness. Specifically, surgical timing (days since injury), sex and age explain 47.6% of the variability in ACL-RSI scores measured 3 to 6-months across studies. Shorter time between injury and surgery, female sex (proportion in the study) and older average age were related to higher average scores. While this is a considerable amount of variability explained early, this relationship diminished and changed 12–24 months after ACLR, with only 27% of variance explained. Further, female sex (proportion in the study) and older average age were then negatively related to ACL-RSI scores. While speculative, it is possible that female sex is related to higher scores due to the considerable focus on prevention of second ACL tears in females. Older age may suggest more mature coping skills through experience, and shorter time from injury to surgery may provide patients with a sense that they are already on the road to recovery. Considering the weak evidence found in this review (due to high risk of bias) and the issues with translating a summary level meta-regression to patient level decisions, clinicians can cautiously consider those who had longer time from injury to surgery, are older and are female as being more prone to reduced ACL-RSI scores 12–24 months after ACLR.

### Clinical Implications

The results of this review have several important implications. Clinicians should monitor ACL-RSI scores across the continuum of care and can compare their patient to the pooled values contained in this review. Clinicians should be aware that psychological readiness tends to plateau from 6 to 24-months after surgery. If this occurs, clinicians should consider further assessment, and actively engage the participant in conversations about the specific ACL-RSI questions (i.e., specific line items or domains) items that they struggle with and develop targeted interventions for each participant.

### Research Recommendations

This review identified that the overwhelming majority of studies measuring ACL-RSI scores were at high risk of bias, especially for selection bias, confounding and missing data. Researchers must be cognizant of these common issues when designing future studies, with active strategies built in to avoid or overcome them. Many of these issues can be overcome by following common reporting guidelines (e.g., STROBE)[122] and engaging statisticians at the outset to plan statistical analysis plans that account for confounding and data imputation procedures. Further, future research should build on this review and assess how age, sex, and time from injury to surgery may predict ACL-RSI scores at important time points after ACLR.

### Limitations

There are several limitations that should be acknowledged. The search end date for this manuscript was March 2022. There is a possibility that papers have been published since the end date of our search. Interpretation of the findings of this systematic review should be adjusted accordingly. Our initial protocol was designed to obtain individual participant data. We were unable to obtain enough data for this to be viable, thus we had to perform an aggregate data meta-analysis. Aggregate data reduced our ability to overcome the many limitations noted (i.e., confounding and missing data). The ACL-RSI scores should not be considered individual trajectories based on longitudinal testing as all study designs (e.g., descriptive study, single time-point) were included in the analysis. In addition, inferences based on the responsiveness to specific treatment cannot be made due to most studies being observational. The analysis based on treatment approach (i.e., early, delayed etc.) is subject to misclassification biases because study level average values were used for classification. Thus, participants who had a delayed ACLR could have been included in the early category and vice-versa. Thus, comparisons between early and delayed approaches can be biased towards the null. This review also included both primary and secondary ACL injuries, however we did not comment on any differences as this was not an explicit objective.

## Conclusion

There is weak evidence that ACL-RSI scores improved from pre-ACLR to 3 to 6-months post-ACLR, but then remained constant until 2 to 5-years post ACLR, where they were the highest (~ 70/100). There were no clinically relevant differences in ACL-RSI scores between studies assessing individuals who had an early or a delayed ACLR. Older age, female sex and longer time from injury to surgery (days) may be associated with lower ACL-RSI scores 1 to 2 years after ACLR. Clinicians should be alert to these individuals and provide early intervention to improve psychological readiness to RTS. Clinicians should monitor psychological readiness over time after ACL injury, as well as work with the patient to identify individualized strategies to address this, especially during periods of score plateau (6 to 24 months). Researchers are encouraged to build on this systematic review and directly compare ACL-RSI scores between individuals who undergo rehabilitation alone versus ACLR and perform high quality studies that allow for further identification of prognostic factors and allow more confident conclusions to be made.

### Supplementary Information

**Additional file 1: Appendix 1.** Systematic search.** Appendix 2.** Author contacts.** Appendix 3.** Risk of bias judgements.** Appendix 4.** Funnel plot.** Appendix 5.** Meta-regression models.

## Data Availability

All data used for this study are provided in the manuscript and supplemental files.

## Abbreviations

ACL: Anterior cruciate ligament
ACLR: Anterior cruciate ligament reconstruction
ACLRSI: Anterior Cruciate Ligament-Return to Sport after Injury scale
CI: Confidence interval
RTS: Return to sport
TSK: Tampa Scale of Kinesiophobia
KSES: Knee-Self Efficacy Scale
FABQ: Fear Avoidance Beliefs Questionnaire
RoB: Risk of bias
RoB 2: Cochrane Risk of Bias tool for randomized trials
ROBINS-I: Risk of Bias tool In Non-randomized Studies of Interventions
RoBANS: Risk of Bias Assessment tool for Nonrandomized Studies
