# Supplementary material for: Anterior Cruciate Ligament Return to Sport after Injury Scale (ACL-RSI) Scores over Time After Anterior Cruciate Ligament Reconstruction: A Systematic Review with Meta-analysis
Source: Sports Med Open. 2024 Apr 30;10:49. doi: 10.1186/s40798-024-00712-w (PMC11061071; doi:10.1186/s40798-024-00712-w)
Supplement: Supplementary file 1 — Additional file 1: Appendix 1. Systematic search. Appendix 2. Author contacts. Appendix 3. Risk of bias judgements. Appendix 4. Funnel plot. Appendix 5. Meta-regression models. [file 40798_2024_712_MOESM1_ESM.pdf]

Appendix 1. Systematic Search

Database (including vendor/platform): Medline (via Ovid)

Set #	Terms	Results
1	exp Anterior Cruciate Ligament/ OR exp Ligaments, Articular/ OR exp Anterior Cruciate Ligament Injuries/ OR exp Anterior Cruciate Ligament Reconstruction/	35289
2	("anterior cruciate ligaments" or ACL or ACLR or "Anterior Cruciate Ligament").ti,ab.	23481
3	1 OR 2	43786
4	exp Fear/ OR exp Self Efficacy/	52699
5	(fear or "Self-efficacy" or "Tampa Scale for Kinesiophobia" or TSK or "ACL-QOL" or ACLQOL or "Anterior Cruciate Ligament Quality Of Life" or "Anterior Cruciate Ligament Return to Sport After Injury" or "Return to Sport after Injury Scale" or "ACL-RSI" or ACLRSI or "K-SES" or " Fear Avoidance Beliefs" or FABQ or "Psychological Readiness" or Fear or confidence or confident or avoidance or Kinesiophobia).ti,ab.	656049
6	4 OR 5	674925
7	3 AND 6	1221
8	not (systematic review.pt OR meta-analysis.pt OR case reports.pt OR editorial.pt OR letter.pt OR comment.pt) not (exp animals/ not exp humans/)	1013

Database (including vendor/platform): Embase (via Elsevier)

Set #	Terms	Results
1	'anterior cruciate ligament injuries'/exp OR 'anterior cruciate ligament injuries':ti,ab OR 'joint ligament'/exp OR 'joint ligament':ti,ab OR 'anterior cruciate ligament'/exp OR 'anterior cruciate ligament':ti,ab OR 'anterior cruciate ligament reconstruction'/exp OR 'anterior cruciate ligament reconstruction':ti,ab OR 'acl'/exp OR acl:ti,ab OR aclr:ti,ab	49,658
2	'fear'/exp OR Fear:ti,ab OR confidence:ti,ab OR confident:ti,ab OR avoidance:ti,ab OR Kinesiophobia:ti,ab OR "Self-efficacy":ti,ab OR "Tampa Scale for Kinesiophobia":ti,ab OR TSK:ti,ab OR "ACL-QOL":ti,ab OR ACLQOL:ti,ab OR 'Anterior Cruciate Ligament Quality Of Life':ti,ab OR 'Anterior Cruciate Ligament Return to Sport After Injury':ti,ab OR 'Return to Sport after Injury Scale':ti,ab OR "ACL-RSI":ti,ab OR ACLRSI:ti,ab OR "K-SES":ti,ab OR 'Fear Avoidance Beliefs':ti,ab OR FABQ:ti,ab OR 'Psychological Readiness':ti,ab	1,033,342
3	1 AND 2	1,664
4	NOT ('case report'/exp OR 'case study'/exp OR 'editorial'/exp OR [editorial]/lim OR 'letter'/exp OR [letter]/lim OR 'note'/exp OR [note]/lim OR [conference abstract]/lim OR 'conference abstract'/exp OR 'conference abstract'/it OR 'systematic review':ti OR 'meta-analysis':ti OR 'systematic review'/exp OR 'meta analysis'/exp) AND [humans]/lim	1,059

Database (including vendor/platform): CINAHL (via EBSCO)

Set #	Terms	Results
1	(MH "Anterior Cruciate Ligament") OR (MH "Anterior Cruciate Ligament Reconstruction") OR (MH "Anterior Cruciate Ligament Injuries") OR TI(“anterior cruciate ligament” OR “anterior cruciate ligaments” OR ACL OR ACLR) OR AB(“anterior cruciate ligament” OR “anterior cruciate ligaments” OR ACL OR ACLR)	14,559
2	(MH "Fear+") OR (MH "Self-Efficacy") OR TI (fear OR confidence OR confident OR avoidance OR Kinesiophobia OR "Self-efficacy" OR "Tampa Scale for Kinesiophobia" OR TSK OR "ACL-QOL" OR ACLQOL OR "Anterior Cruciate Ligament Quality Of Life" OR "Anterior Cruciate Ligament Return to Sport After Injury" OR "Return to Sport after Injury Scale" OR "ACL-RSI" OR ACLRSI OR "K-SES" OR "Fear Avoidance Beliefs" OR FABQ OR "Psychological Readiness") OR AB (fear OR confidence OR confident OR avoidance OR Kinesiophobia OR "Self-efficacy" OR "Tampa Scale for Kinesiophobia" OR TSK OR "ACL-QOL" OR ACLQOL OR "Anterior Cruciate Ligament Quality Of Life" OR "Anterior Cruciate Ligament Return to Sport After Injury" OR "Return to Sport after Injury Scale" OR "ACL-RSI" OR ACLRSI OR "K-SES" OR "Fear Avoidance Beliefs" OR FABQ OR "Psychological Readiness")	**273,940**
3	1 and 2	711
4	S3 NOT PT (Case study OR Abstract OR Commentary OR Editorial OR Letter OR Meta Analysis OR Meta Synthesis OR Systematic Review)	578

Database (including vendor/platform): Web of Science (via Clarivate)

Set #	Terms	Results
1	TS=(“anterior cruciate ligament” OR “anterior cruciate ligaments” OR ACL OR ACLR)	28,678
2	TS=(fear OR confidence OR confident OR avoidance OR Kinesiophobia OR "Self-efficacy" OR "Tampa Scale for Kinesiophobia" OR TSK OR "ACL-QOL" OR ACLQOL OR "Anterior Cruciate Ligament Quality Of Life" OR "Anterior Cruciate Ligament Return to Sport After Injury" OR "Return to Sport after Injury Scale" OR "ACL-RSI" OR ACLRSI OR "K-SES" OR "Fear Avoidance Beliefs" OR FABQ OR "Psychological Readiness")	893,851
3	1 and 2	1,039
4	Refined by: [excluding] DOCUMENT TYPES: (EDITORIAL MATERIAL OR REVIEW OR MEETING ABSTRACT OR LETTER OR PROCEEDINGS PAPER)	871

Database (including vendor/platform): Scopus (via Elsevier)

Set #	Terms	Results

1	TITLE-ABS-KEY ("anterior cruciate ligament" OR "anterior cruciate ligaments" OR acl OR aclr)	37,518
2	TITLE-ABS-KEY(fear OR confidence OR confident OR avoidance OR Kinesiophobia OR "Self-efficacy" OR "Tampa Scale for Kinesiophobia" OR TSK OR "ACL-QOL" OR ACLQOL OR "Anterior Cruciate Ligament Quality Of Life" OR "Anterior Cruciate Ligament Return to Sport After Injury" OR "Return to Sport after Injury Scale" OR "ACL-RSI" OR ACLRSI OR "K-SES" OR "Fear Avoidance Beliefs" OR FABQ OR "Psychological Readiness")	1,103,196
3	1 and 2	1,224
4	AND (EXCLUDE (DOCTYPE , "re") OR EXCLUDE (DOCTYPE , "cp") OR EXCLUDE (DOCTYPE , "no") OR EXCLUDE (DOCTYPE , "ch") OR EXCLUDE (DOCTYPE , "le") OR EXCLUDE (DOCTYPE , "ed") OR EXCLUDE (DOCTYPE , "cr"))	1,036

Database (including vendor/platform): Cochrane Central Register of Controlled Trials (ClinicalTrials.gov and WHO's International Clinical Trials Registry Platform)

Set #	Terms	Results
1	[mh "Anterior Cruciate Ligament"] OR [mh "Ligaments, Articular"] OR [mh "Anterior Cruciate Ligament Injuries"] OR [mh "Anterior cruciate ligament reconstruction"] OR "anterior cruciate ligament":ti,ab OR "anterior cruciate ligaments":ti,ab OR acl:ti,ab OR aclr:ti,ab	3,173
2	[mh "Fear"] OR [mh "Self Efficacy"] OR Fear:ti,ab OR confidence:ti,ab OR confident:ti,ab OR avoidance:ti,ab OR Kinesiophobia:ti,ab OR "Self-efficacy":ti,ab OR "Tampa Scale for Kinesiophobia":ti,ab OR TSK:ti,ab OR "ACL-QOL":ti,ab OR ACLQOL:ti,ab OR "Anterior Cruciate Ligament Quality Of Life":ti,ab OR "Anterior Cruciate Ligament Return to Sport After Injury":ti,ab OR "Return to Sport after Injury Scale":ti,ab OR "ACL-RSI":ti,ab OR ACLRSI:ti,ab OR "K-SES":ti,ab OR "Fear Avoidance Beliefs":ti,ab OR FABQ:ti,ab OR "Psychological Readiness":ti,ab	92,890
3	1 and 2	145
4	Limit to Trials	132

Database (including vendor/platform): SportDiscus (via EbscoHost)

Set #	Terms	Results
1	(DE "ANTERIOR cruciate ligament" OR DE "ANTERIOR cruciate ligament injuries" OR DE "ANTERIOR cruciate ligament surgery" OR DE "ANTERIOR cruciate ligament transplantation") OR TI(“anterior cruciate ligament” OR “anterior cruciate ligaments” OR ACL OR ACLR) OR AB(“anterior cruciate ligament” OR “anterior cruciate ligaments” OR ACL OR ACLR)	13,362
2	(DE "FEAR") OR (DE "SELF-efficacy") OR TI (fear OR confidence OR confident OR avoidance OR Kinesiophobia OR "Self-efficacy" OR "Tampa Scale for Kinesiophobia" OR TSK OR "ACL-QOL" OR ACLQOL OR "Anterior Cruciate Ligament Quality Of Life" OR "Anterior Cruciate Ligament Return	48,429

	to Sport After Injury" OR "Return to Sport after Injury Scale" OR "ACL-RSI" OR ACLRSI OR "K-SES" OR "Fear Avoidance Beliefs" OR FABQ OR "Psychological Readiness") OR AB (fear OR confidence OR confident OR avoidance OR Kinesiophobia OR "Self-efficacy" OR "Tampa Scale for Kinesiophobia" OR TSK OR "ACL-QOL" OR ACLQOL OR "Anterior Cruciate Ligament Quality Of Life" OR "Anterior Cruciate Ligament Return to Sport After Injury" OR "Return to Sport after Injury Scale" OR "ACL-RSI" OR ACLRSI OR "K-SES" OR "Fear Avoidance Beliefs" OR FABQ OR "Psychological Readiness")	
3	1 and 2	508
4	S3 NOT PT (Conference paper OR Conference proceeding OR Review)	508

Appendix 2. Author Contacts

Study	Information Clarified	Outcome
Alzhrani 2022	No	ACL treatment type = Unclear
Ardern, 2014	Yes	ACL treatment type = Delayed ACLR w/o prior exercise therapy
Betsch 2021	No	ACL treatment type = Unclear
Blakeney, 2018	No	ACL treatment type = Unclear
Bohu, 2015	No	ACL treatment type = Unclear
Bortone 2021	No	ACL treatment type = Unclear & Excluded from meta-analysis
Burland, 2018	Yes	ACL treatment type = Early ACLR
Burland, 2020	Yes	ACL treatment type = Early ACLR
Byrne 2021	No	ACL treatment type = Unclear
Chen, 2017	No	ACL treatment type = Unclear
Cusumano 2021	Yes	ACL-RSI Mean/SD Added & ACL treatment type = Delayed ACLR without prior exercise therapy
Falstrom 2016	Yes	ACL treatment type = Early ACLR & Unclear
Gokeler, 2017	No	ACL treatment type = Unclear
Goto 2022	No	ACL treatment type = Unclear
Ha, 2019	No	ACL treatment type = Unclear
Harput, 2016	No	ACL treatment type = Unclear
Harput, 2017	No	ACL treatment type = Unclear
Hart, 2020	Yes	ACL treatment type = Delayed ACLR w/o prior exercise therapy
Hirohata, 2020	No	ACL treatment type = Unclear
Jia, 2018	No	ACL treatment type = Unclear
Kim 2021	No	ACL treatment type = Unclear
Klasan, 2020	Yes	ACL treatment type = Early ACLR; ACL-RSI Mean/SD Added
Kuenze, 2021	No	ACL treatment type = Unclear
Kvist, 2013	Yes	ACL treatment type = Delayed ACLR w/o prior exercise therapy
Lee 2021	No	ACL treatment type = Unclear
Lefevre, 2017	No	Excluded
Ling, 2019	No	ACL treatment type = Unclear
Martini 2022	Yes	ACL-RSI Mean/SD Added

McPherson, 2019	No	ACL treatment type = Unclear
Meierbachtol, 2018	Yes	ACL treatment type = Early ACLR
Meierbachtol, 2020	Yes	ACL treatment type = Early ACLR
Monaco 2022	Yes	ACL treatment type = Early ACLR
Nagelli, 2019	Yes	ACL treatment type = Early ACLR
O'Connor, 2020	No	Excluded
Oliveira Silva, 2018	No	Excluded
Peebles, 2021	Yes	ACL treatment type = Early ACLR
Phelan, 2019	No	ACL treatment type = Unclear
Presley, 2021	No	ACL treatment type = Unclear
Rhim, 2020	No	ACL treatment type = Unclear
Rosso, 2018	No	ACL treatment type = Unclear
Sadeqi, 2018	No	ACL treatment type = Unclear
Sala-Barat, 2020	No	ACL-RSI Mean/SD Added
Salatkaite 2021	No	ACL treatment type = Unclear
Salatkaite, 2019	No	ACL treatment type = Unclear
Slagers, 2017	No	ACL treatment type = Unclear
Slagers, 2019	No	ACL treatment type = Unclear
Thiebat, 2021	No	ACL treatment type = Unclear
Toale, 2021	Yes	ACL treatment type = Early ACLR & Delayed ACLR w/o prior exercise therapy
Tortoli, 2020	No	ACL treatment type = Unclear
Webster 2021	No	ACL treatment type = Unclear
Webster, 2008	Yes	ACL treatment type = Early ACLR
Webster, 2018 ("Development…Short Form…")	Yes	ACL treatment type = Early ACLR
Webster, 2018 ("Factors…Psych Readiness…")	Yes	ACL treatment type = Early ACLR
Webster, 2019	Yes	ACL treatment type = Early ACLR
Webster, 2020	Yes	ACL treatment type = Unclear;<br>ACL-RSI Mean/SD Added
Welling, 2018	No	ACL treatment type = Unclear
Wilson, 2018	No	ACL treatment type = Unclear
Winkler, 2021	Yes	ACL treatment type = Early ACLR
Zanovello, 2017	No	ACL treatment type = Unclear
Zarzycki 2021	No	ACL treatment type = Unclear
Zarzycki, 2018	No	ACL treatment type = Unclear

Appendix 3. Risk of Bias Judgements

Risk of Bias Assessment Tool for Nonrandomized Studies (RoBANS)

ACL Injury Treatment Category	Study	Participant Selection	Confounding	Exposure Measurement	Blinding of Outcome Assessors	Missing Data	Selective Outcome Reporting	Overall
Early ACLR	Ardern et al. 2015 [1]	Low	N/A	Low	Low	High	Low	**Low**
	Burland et al. 2018 [2]	High	N/A	Low	Low	Low	Low	**Low**
	Burland et al. 2020 [3]	High	N/A	Low	Low	Low	High	**High**
	Culvenor et al. 2016 [4]	Low	Low	Low	Unclear	Unclear	Low	**High**
	Ebert et al. 2021 [5]	Low	High	Low	N/A	Unclear	Low	**High**
	Fones et al. 2020 [6]	Low	Low	Low	High	Unclear	Low	**High**
	Johnston et al. 2021 [7]	High	Low	Low	Unclear	Unclear	High	**High**
	Kitaguchi et al. 2020 [8]	Low	N/A	Low	Unclear	Low	High	**High**
	Klasan et al. 2021 [9]	Low	N/A	Low	N/A	Unclear	High	**High**
	Langford et al. 2009 [10]	High	High	Low	Unclear	Unclear	Low	**High**
	Lepley et al. 2018 [11]	High	High	Low	N/A	Unclear	Low	**High**
	Lisee et al. 2020 [12]	Low	High	Low	High	Unclear	Low	**High**
	Martini et al. 2022 [13]	Low	High	Low	Unclear	Low	High	**High**
	Meierbachtol et al. 2020 [14]	High	N/A	Low	N/A	Unclear	Low	**High**
	Monaco et al. 2022 [15]	Low	N/A	Low	N/A	Low	Low	**Low**
	Nagelli et al. 2019 [16]	High	High	Low	N/A	Unclear	Low	**High**
	Ohji et al. 2021 [17]	High	High	Low	Unclear	Low	Low	**High**

	Ohji et al. 2021 [18]	Low	N/A	Low	Unclear	Unclear	Low	**High**
	Panisset et al. 2019 [19]	Low	High	Low	Unclear	Unclear	Low	**High**
	Peebles et al. 2021 [20]	High	High	Low	N/A	Unclear	Low	**High**
	Sala-Barat et al. 2020 [21]	High	High	Low	Unclear	Unclear	High	**High**
	Vermeijden et al. 2021 [22]	High	N/A	Low	Unclear	Low	Low	**Low**
	Webster et al 2008 [23]	Unclear	N/A	Low	Low	Unclear	Low	**High**
	Webster et al. 2018 [24]	Unclear	N/A	Low	High	Unclear	Low	**High**
	Webster et al. 2018 [25]	Low	Low	Low	Low	Low	Low	**Low**
	Webster et al. 2019 [26]	High	N/A	Low	Low	High	Low	**High**
	Winkler et al. 2021 [27]	Low	High	Low	Low	High	High	**High**
Delayed ACLR without prior exercise therapy	Ardern et al. 2014 [28]	Low	N/A	Low	Low	Low	Low	**Low**
	Ardern et al. 2013 [29]	Low	N/A	Low	Low	Low	Low	**Low**
	Beischer et al. 2019 [30]	Low	High	Low	Low	High	Low	**High**
	Faleide et al. 2021 [31]	High	N/A	Low	Low	Unclear	Low	**High**
	Fayard et al. 2019 [32]	High	N/A	Low	N/A	Unclear	High	**High**
	Hart et al. 2020 [33]	Low	N/A	Low	N/A	Unclear	Low	**Low**
	Kvist et al. 2013 [34]	Low	N/A	Low	Unclear	Low	Low	**Low**
	Pioger et al. 2021 [35]	Low	High	Low	Unclear	High	Low	**High**
	Piussi et al. 2020 [36]	Low	High	Low	Low	Unclear	High	**High**
	Rayes et al. 2022 [37]	Low	High	Low	Unclear	High	Low	**High**

Delayed ACLR	Toanen et al. 2017 [38]	Low	High	Low	N/A	Unclear	Low	**High**
Rehabilitation Only	Fayard et al. 2019 [39]	Low	N/A	Low	Unclear	High	Low	**High**
Early ACLR & Delayed ACLR without prior exercise therapy	Kostyun et al. 2021 [40]	High	High	Low	Low	Low	Low	**High**
	Toale et al. 2021 [41]	Low	High	Low	Unclear	High	Low	**High**
Early ACLR & Rehabilitation Only	Ehlinger et al. 2021 [42]	Low	High	Low	Unclear	Unclear	Low	**High**
Early ACLR & Unclear	Faltstrom et al. 2016 [43]	Low	N/A	Low	Low	Low	Low	**Low**
Unclear	Alzrani et al. 2022 [44]	High	N/A	High	N/A	Low	Low	**High**
	Betsch et al. 2021 [45]	Low	High	Low	N/A	Low	Low	**Low**
	Blakeney et al. 2018 [46]	High	N/A	Unclear	Unclear	Unclear	Low	**High**
	Bohu et al. 2015 [47]	Low	N/A	Low	Low	Low	Low	**Low**
	Bortone et al. 2021 [48]	High	High	Low	Unclear	Unclear	Low	**High**
	Byrne et al. 2021 [49]	High	High	Low	Unclear	Unclear	Low	**High**
	Chen et al. 2017[50]	High	N/A	Low	N/A	Low	Low	**Low**
	Gokeler et al. 2017 [51]	Unclear	High	Low	N/A	Unclear	High	**High**
	Goto et al. 2022 [52]	High	N/A	Low	Unclear	Low	Low	**High**
	Ha et al. 2019 [53]	Unclear	N/A	Low	Unclear	Unclear	Low	**High**
	Harput et al. 2016 [54]	Unclear	High	Unclear	N/A	Unclear	High	**High**
	Harput et al. 2017 [55]	Unclear	N/A	Low	N/A	Low	Low	**Low**
	Hirohata et al. 2020 [56]	High	High	Unclear	N/A	Low	Low	**High**

	Jia et al. 2018 [57]	Low	N/A	Low	Unclear	Unclear	Low	**High**
	Kim et al. 2022 [58]	Low	High	Low	Unclear	Unclear	Low	**High**
	Kuenze et al. 2021 [59]	Unclear	Low	High	Unclear	Low	Low	**High**
	Lee et al. 2021 [60]	Low	High	Low	Unclear	Unclear	Low	**High**
	McPherson et al. 2019 [61]	High	High	Low	Low	High	Low	**High**
	Phelan et al. 2019 [62]	Low	Low	Low	High	Unclear	Low	**High**
	Presley et al. 2021 [63]	Low	Low	Low	Unclear	Unclear	Low	**High**
	Rosso et al. 2018 [64]	Unclear	Low	Low	N/A	Unclear	Low	**High**
	Sadeqi et al. 2018 [65]	Low	High	Low	Low	Unclear	Low	**High**
	Salatkaite et al. 2021 [66]	High	N/A	Unclear	Unclear	Unclear	Low	**High**
	Salatkaite et al. 2019 [67]	High	N/A	Unclear	Unclear	Low	Low	**High**
	Slagers et al. 2017 [68]	Low	N/A	Low	N/A	Unclear	Low	**Low**
	Slagers et al. 2019 [69]	Low	High	Low	N/A	Low	Low	**Low**
	Thiebat et al. 2022 [70]	Low	High	Low	N/A	Low	Low	**High**
	Tortoli et al. 2022 [71]	High	N/A	Low	High	Unclear	Low	**High**
	Webster et al. 2021 [72]	Low	High	Low	N/A	Low	Low	**Low**
	Welling et al. 2018 [73]	High	Low	Low	N/A	Low	Low	**Low**
	Willson et al. 2018 [74]	Low	High	Low	N/A	Unclear	Low	**High**
	Zanovello et al. 2017 [75]	Low	High	Low	N/A	Unclear	High	**High**
	Zarzycki et al. 2021 [76]	High	High	Low	Unclear	Low	Low	**High**

	Zarzycki et al. 2018 [77]	High	High	Low	Unclear	Low	High	**High**

Risk of Bias Tool in Non-randomized Studies of Interventions (ROBINS-I)

ACL Injury Treatment Category	Study	Confounding	Participant Selection	Intervention Classification	Deviations from Intended Interventions	Missing Data	Outcome Measurement	Selection of Reported Result	Overall
Early ACLR	Meierbachtol et al. 2018 [78]	Serious	Moderate	Moderate	Low	Unclear	Low	Low	**Critical**
Early ACLR & Delayed ACLR without prior exercise therapy	Muller et al. 2015 [79]	Serious	Moderate	Low	Low	Unclear	Low	Serious	**Critical**

Cochrane Risk of Bias Tool for Randomized Studies (ROB2)

ACL Injury Treatment Category	Study	Randomization Process		Deviations from Intended Interventions		Missing Data	Outcome Measurement	Selection of Reported Result	Overall
		Sequence Generation	Allocation Concealment	Blinding of Participants	Blinding of Outcome Assessors				
Delayed ACLR without prior exercise therapy	Cusumano et al. 2021 [80]	Some Concerns	Unclear	Low	Low	Unclear	Low	High	**High**
Early ACLR	Sanborn et al. 2022 [81]	Low	Unclear	Low	Some Concerns	Low	Unclear	Low	**High**
Unclear	Rhim et al. 2020 [82]	Some Concerns	Unclear	Unclear	Unclear	Unclear	Low	High	**High**
	Ling et al. 2019 [83]	Low	Unclear	Unclear	Unclear	Unclear	Low	Some Concerns	**High**

ACL (anterior cruciate ligament), ACLR (anterior cruciate ligament reconstruction)

Appendix 4. Funnel Plot. Funnel Plot to assess for publication bias for the primary meta-analysis pooling ACL-RSI scores. The x-axis reports the mean of the ACL-RSI scores for each included study. The y-axis reports the standard error for each study. There is no concerns for publication bias based on symmetrical funnel plot

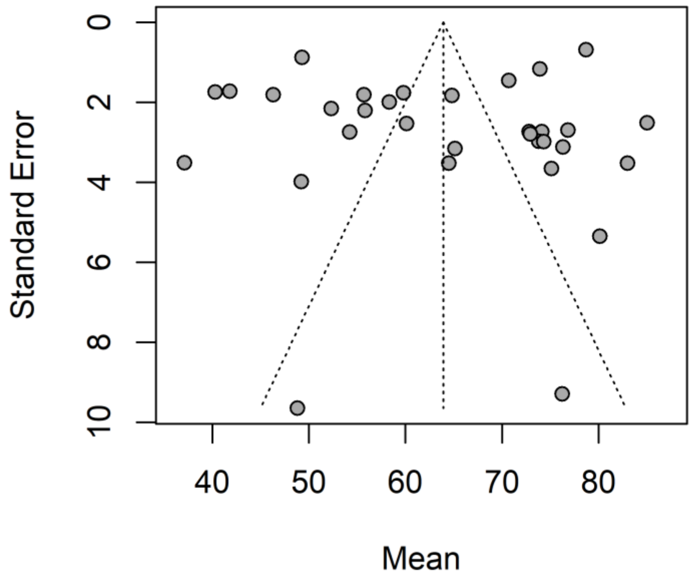

Appendix 5. Meta-Regression Models

A. Final Model Including Age, Sex and Time from Injury to Treatment for ACL-RSI measured between 3 to 6 months after ACLR

Mixed-Effects Model (k = 9; tau^2 estimator: REML)
tau^2 (estimated amount of residual heterogeneity): 35.66 (SE = 29.18)
tau (square root of estimated tau^2 value): 5.97
I^2 (residual heterogeneity / unaccounted variability): 80.3%
H^2 (unaccounted variability / sampling variability): 5.07
R^2 (amount of heterogeneity accounted for): 47.59%
Test for Residual Heterogeneity:
QE(df = 5) = 24.43, p= 0.0002
Test of Moderators (coefficients 2:4):
QM(df = 3) = 8.97, p= 0.030
Model Results:

Factor	Estimate	Standard Error	z-value	p-value	95% CI
Intercept	46.6	15.6	3.0	0.003	16.0, 77.2
Female (%)	23.3	19.7	1.2	0.24	-15.2, 61.8
Age (years)	1.3	0.7	1.9	0.05	-.01, 2.6
Time from injury to Tx (days)	-0.3	0.09	-2.9	0.003	-0.5, -0.09

Tx (treatment), CI (confidence interval)

B. Final Model Including Age, Sex and Time from Injury to Treatment for ACL-RSI measured between 1 to 2 years after ACLR

Mixed-Effects Model (k = 11; tau^2 estimator: REML)
tau^2 (estimated amount of residual heterogeneity): 61.32 (SE = 41.5)
tau (square root of estimated tau^2 value): 7.8
I^2 (residual heterogeneity / unaccounted variability): 92.5%
H^2 (unaccounted variability / sampling variability): 13.28
R^2 (amount of heterogeneity accounted for): 26.97%
Test for Residual Heterogeneity: QE(df = 6) = 87.0, p< .0001
Test of Moderators (coefficients 2:4): QM(df = 3) = 6.03, p= 0.11

Model Results:

Factor	Estimate	Standard Error	z-value	p-value	95% CI
Intercept	157.2	45.9	3.4	0.0006	67.4, 247.1
Female (%)	-59.7	45.3	-1.3	0.2	-148.4, 29.0
Age (years)	-2.7	1.1	-2.3	0.02	-4.9, -0.4
Time from injury to Tx (days)	-0.0009	0.03	-0.03	0.97	-0.05, 0.05

Tx (treatment), CI (confidence interval)

References

1. Ardern CL, Taylor NF, Feller JA, Whitehead TS, Webster KE. Sports Participation 2 Years After Anterior Cruciate Ligament Reconstruction in Athletes Who Had Not Returned to Sport at 1 Year. The American Journal of Sports Medicine. 2015;43(4):848-56.
2. Burland JP, Kostyun RO, Kostyun KJ, Solomito M, Nissen C, Milewski MD. Clinical Outcome Measures and Return-to-Sport Timing in Adolescent Athletes After Anterior Cruciate Ligament Reconstruction. J Athl Train. 2018 May;53(5):442-51.
3. Burland JP, Howard JS, Lepley AS, DiStefano LJ, Lepley LK, Frechette L. What Are Our Patients Really Telling Us? Psychological Constructs Associated With Patient-Reported Outcomes After Anterior Cruciate Ligament Reconstruction. J Athl Train. 2020 Jul 1;55(7):707-16.
4. Culvenor AG, Collins NJ, Vicenzino B, Cook JL, Whitehead TS, Morris HG, Crossley KM. Predictors and effects of patellofemoral pain following hamstring-tendon ACL reconstruction. J Sci Med Sport. 2016 Jul;19(7):518-23.
5. Ebert JR, Edwards P, Preez LD, Furzer B, Joss B. Knee extensor strength, hop performance, patient-reported outcome and inter-test correlation in patients 9-12 months after anterior cruciate ligament reconstruction. Knee. 2021 Jun;30:176-84.
6. Fones L, Kostyun RO, Cohen AD, Pace JL. Patient-Reported Outcomes, Return-to-Sport Status, and Reinjury Rates After Anterior Cruciate Ligament Reconstruction in Adolescent Athletes: Minimum 2-Year Follow-up. Orthop J Sports Med. 2020 Nov;8(11):2325967120964471.
7. Johnston PT, Feller JA, McClelland JA, Webster KE. Strength deficits and flexion range of motion following primary anterior cruciate ligament reconstruction differ between quadriceps and hamstring autografts. J ISAKOS. 2021 Mar;6(2):88-93.
8. Kitaguchi T, Tanaka Y, Takeshita S, Tsujimoto N, Kita K, Amano H, et al. Importance of functional performance and psychological readiness for return to preinjury level of sports 1 year after ACL reconstruction in competitive athletes. Knee Surg Sports Traumatol Arthrosc. 2020 Jul;28(7):2203-12.
9. Klasan A, Putnis SE, Grasso S, Kandhari V, Oshima T, Parker DA. Tegner level is predictive for successful return to sport 2 years after anterior cruciate ligament reconstruction. Knee Surg Sports Traumatol Arthrosc. 2021 Sep;29(9):3010-6.
10. Langford JL, Webster KE, Feller JA. A prospective longitudinal study to assess psychological changes following anterior cruciate ligament reconstruction surgery. Br J Sports Med. 2009 May;43(5):377-81.
11. Lepley AS, Pietrosimone B, Cormier ML. Quadriceps Function, Knee Pain, and Self-Reported Outcomes in Patients With Anterior Cruciate Ligament Reconstruction. J Athl Train. 2018 Apr;53(4):337-46.
12. Lisee CM, DiSanti JS, Chan M, Ling J, Erickson K, Shingles M, Kuenze CM. Gender Differences in Psychological Responses to Recovery After Anterior Cruciate Ligament Reconstruction Before Return to Sport. J Athl Train. 2020 Oct 1;55(10):1098-105.
13. Martini A, Ayala A, Lechable M, Rannou F, Lefevre-Colau MM, Nguyen C. Determinants of apprehension to return to sport after reconstruction of the anterior cruciate ligament: an

exploratory observational retrospective study. BMC Sports Sci Med Rehabil. 2022 Mar 15;14(1):37.

14. Meierbachtol A, Obermeier M, Yungtum W, Bottoms J, Paur E, Nelson BJ, et al. Injury-Related Fears During the Return-to-Sport Phase of ACL Reconstruction Rehabilitation. Orthop J Sports Med. 2020 Mar;8(3):2325967120909385.

15. Monaco E, Pisanu G, Carrozzo A, Giuliani A, Conteduca J, Oliviero M, et al. Translation, cross-cultural adaptation, and validation of the Italian version of the anterior cruciate ligament-return to sport after injury (ACL-RSI) scale and its integration into the K-STARTS test. J Orthop Traumatol. 2022 Feb 21;23(1):11.

16. Nagelli CV, Webster KE, Di Stasi S, Wordeman SC, Hewett TE. The association of psychological readiness to return to sport after anterior cruciate ligament reconstruction and hip and knee landing kinematics. Clin Biomech (Bristol, Avon). 2019 Aug;68:104-8.

17. Ohji S, Aizawa J, Hirohata K, Mitomo S, Ohmi T, Jinno T, et al. Athletic identity and sport commitment in athletes after anterior cruciate ligament reconstruction who have returned to sports at their pre-injury level of competition. BMC Sports Sci Med Rehabil. 2021 Apr 7;13(1):37.

18. Ohji S, Aizawa J, Hirohata K, Ohmi T, Mitomo S, Koga H, Yagishita K. The psychological readiness to return to sports of patients with anterior cruciate ligament reconstruction preoperatively and 6 months postoperatively. Phys Ther Sport. 2021 Jul;50:114-20.

19. Panisset JC, Gonzalez JF, de Lavigne C, Ode Q, Dejour D, Ehlinger M, et al. ACL reconstruction in over-50 year-olds: Comparative study between prospective series of over-50 year-old and under-40 year-old patients. Orthop Traumatol Surg Res. 2019 Dec;105(8S):S259-S65.

20. Peebles AT, Savla J, Ollendick TH, Queen RM. Landing Asymmetry Is Associated with Psychological Factors after Anterior Cruciate Ligament Reconstruction. Med Sci Sports Exerc. 2021 Jul 1;53(7):1446-51.

21. Sala-Barat E, Alvarez-Diaz P, Alentorn-Geli E, Webster KE, Cugat R, Tomas-Sabado J. Translation, cross-cultural adaptation, validation, and measurement properties of the Spanish version of the anterior cruciate ligament-return to sport after injury (ACL-RSI-Sp) scale. Knee Surg Sports Traumatol Arthrosc. 2020 Mar;28(3):833-9.

22. Vermeijden HD, van der List JP, DiFelice GS. Acute and delayed anterior cruciate ligament repair results in similar short to mid-term outcomes. Knee. 2021 Mar;29:142-9.

23. Webster KE, Feller JA, Lambros C. Development and preliminary validation of a scale to measure the psychological impact of returning to sport following anterior cruciate ligament reconstruction surgery. Phys Ther Sport. 2008 Feb;9(1):9-15.

24. Webster KE, Feller JA. Development and Validation of a Short Version of the Anterior Cruciate Ligament Return to Sport After Injury (ACL-RSI) Scale. Orthop J Sports Med. 2018 Apr;6(4):2325967118763763.

25. Webster KE, Nagelli CV, Hewett TE, Feller JA. Factors Associated With Psychological Readiness to Return to Sport After Anterior Cruciate Ligament Reconstruction Surgery. American Journal of Sports Medicine. 2018;46(7):1545-50.

26. Webster KE, McPherson AL, Hewett TE, Feller JA. Factors Associated With a Return to Preinjury Level of Sport Performance After Anterior Cruciate Ligament Reconstruction Surgery. Am J Sports Med. 2019 Sep;47(11):2557-62.

27. Winkler PW, Wagala NN, Hughes JD, Lesniak BP, Musahl V. A high tibial slope, allograft use, and poor patient-reported outcome scores are associated with multiple ACL graft failures. Knee Surg Sports Traumatol Arthrosc. 2022 Jan;30(1):139-48.
28. Ardern CL, Taylor NF, Feller JA, Webster KE. Fifty-five per cent return to competitive sport following anterior cruciate ligament reconstruction surgery: an updated systematic review and meta-analysis including aspects of physical functioning and contextual factors. Br J Sports Med. 2014 Nov;48(21):1543-52.
29. Ardern CL, Taylor NF, Feller JA, Whitehead TS, Webster KE. Psychological responses matter in returning to preinjury level of sport after anterior cruciate ligament reconstruction surgery. American Journal of Sports Medicine. 2013;41(7):1549-58.
30. Beischer S, Hamrin Senorski E, Thomee C, Samuelsson K, Thomee R. How Is Psychological Outcome Related to Knee Function and Return to Sport Among Adolescent Athletes After Anterior Cruciate Ligament Reconstruction? The American journal of sports medicine. 2019;47(7):1567-75.
31. Faleide AGH, Magnussen LH, Strand T, Bogen BE, Moe-Nilssen R, Mo IF, et al. The Role of Psychological Readiness in Return to Sport Assessment After Anterior Cruciate Ligament Reconstruction. Am J Sports Med. 2021 Apr;49(5):1236-43.
32. Fayard JM, Wein F, Ollivier M, Paihle R, Ehlinger M, Lustig S, et al. Factors affecting outcome of ACL reconstruction in over-50-year-olds. Orthop Traumatol Surg Res. 2019 Dec;105(8S):S247-S51.
33. Hart HF, Culvenor AG, Guermazi A, Crossley KM. Worse knee confidence, fear of movement, psychological readiness to return-to-sport and pain are associated with worse function after ACL reconstruction. Phys Ther Sport. 2020 Jan;41:1-8.
34. Kvist J, Osterberg A, Gauffin H, Tagesson S, Webster K, Ardern C. Translation and measurement properties of the Swedish version of ACL-Return to Sports after Injury questionnaire. Scand J Med Sci Sports. 2013 Oct;23(5):568-75.
35. Pioger C, Saithna A, Rayes J, Haidar IM, Fradin T, Ngbilo C, et al. Influence of Preoperative Tunnel Widening On the Outcomes of a Single Stage-Only Approach to Every Revision Anterior Cruciate Ligament Reconstruction: An Analysis of 409 Consecutive Patients From the SANTI Study Group. Am J Sports Med. 2021 May;49(6):1431-40.
36. Piussi R, Beischer S, Thomee R, Hamrin Senorski E. Hop tests and psychological PROs provide a demanding and clinician-friendly RTS assessment of patients after ACL reconstruction, a registry study. BMC sports science, medicine & rehabilitation. 2020;12:32.
37. Rayes J, Ouanezar H, Haidar IM, Ngbilo C, Fradin T, Vieira TD, et al. Revision Anterior Cruciate Ligament Reconstruction Using Bone-Patellar Tendon-Bone Graft Combined With Modified Lemaire Technique Versus Hamstring Graft Combined With Anterolateral Ligament Reconstruction: A Clinical Comparative Matched Study With a Mean Follow-up of 5 Years From The SANTI Study Group. Am J Sports Med. 2022 Feb;50(2):395-403.
38. Toanen C, Demey G, Ntagiopoulos PG, Ferrua P, Dejour D. Is There Any Benefit in Anterior Cruciate Ligament Reconstruction in Patients Older Than 60 Years? Am J Sports Med. 2017 Mar;45(4):832-7.
39. Fayard JM, Sonnery-Cottet B, Vrgoc G, O'Loughlin P, de Mont Marin GD, Freychet B, et al. Incidence and Risk Factors for a Partial Anterior Cruciate Ligament Tear Progressing to a

Complete Tear After Nonoperative Treatment in Patients Younger Than 30 Years. Orthop J Sports Med. 2019 Jul;7(7):2325967119856624.
40. Kostyun RO, Burland JP, Kostyun KJ, Milewski MD, Nissen CW. Male and Female Adolescent Athletes' Readiness to Return to Sport After Anterior Cruciate Ligament Injury and Reconstruction. Clin J Sport Med. 2021 Jul 1;31(4):383-7.
41. Toale JP, Hurley ET, Hughes AJ, Withers D, King E, Jackson M, Moran R. The majority of athletes fail to return to play following anterior cruciate ligament reconstruction due to reasons other than the operated knee. Knee Surg Sports Traumatol Arthrosc. 2021 Nov;29(11):3877-82.
42. Ehlinger M, Panisset JC, Dejour D, Gonzalez JF, Paihle R, Favreau H, et al. Anterior cruciate ligament reconstruction in the over-50s. A prospective comparative study between surgical and functional treatment. Orthop Traumatol Surg Res. 2021 Dec;107(8S):103039.
43. Faltstrom A, Hagglund M, Kvist J. Factors associated with playing football after anterior cruciate ligament reconstruction in female football players. Scand J Med Sci Sports. 2016 Nov;26(11):1343-52.
44. Alzhrani M, Alzahrani H, Alshehri YS. Arabic Version of the Short Anterior Cruciate Ligament-Return to Sport After Injury Scale: Translation, Cross-cultural Adaptation, and Validation. Orthop J Sports Med. 2022 Jan;10(1):23259671211066509.
45. Betsch M, Hoit G, Dwyer T, Whelan D, Theodoropoulos J, Ogilvie-Harris D, Chahal J. Postoperative Pain Is Associated With Psychological and Physical Readiness to Return to Sports One-Year After Anterior Cruciate Ligament Reconstruction. Arthrosc Sports Med Rehabil. 2021 Dec;3(6):e1737-e43.
46. Blakeney WG, Ouanezar H, Rogowski I, Vigne G, Guen ML, Fayard JM, et al. Validation of a Composite Test for Assessment of Readiness for Return to Sports After Anterior Cruciate Ligament Reconstruction: The K-STARTS Test. Sports Health. 2018 Nov/Dec;10(6):515-22.
47. Bohu Y, Klouche S, Lefevre N, Webster K, Herman S. Translation, cross-cultural adaptation and validation of the French version of the Anterior Cruciate Ligament-Return to Sport after Injury (ACL-RSI) scale. Knee Surg Sports Traumatol Arthrosc. 2015 Apr;23(4):1192-6.
48. Bortone I, Moretti L, Bizzoca D, Caringella N, Delmedico M, Piazzolla A, Moretti B. The importance of biomechanical assessment after Return to Play in athletes with ACL-Reconstruction. Gait Posture. 2021 Jul;88:240-6.
49. Byrne L, King E, Mc Fadden C, Jackson M, Moran R, Daniels K. The effect of meniscal pathology and management with ACL reconstruction on patient-reported outcomes, strength, and jump performance ten months post-surgery. Knee. 2021 Oct;32:72-9.
50. Chen T, Zhang P, Li Y, Webster K, Zhang J, Yao W, et al. Translation, cultural adaptation and validation of simplified Chinese version of the anterior cruciate ligament return to sport after injury (ACL-RSI) scale. PLoS One. 2017;12(8):e0183095.
51. Gokeler A, Welling W, Zaffagnini S, Seil R, Padua D. Development of a test battery to enhance safe return to sports after anterior cruciate ligament reconstruction. Knee Surg Sports Traumatol Arthrosc. 2017 Jan;25(1):192-9.
52. Goto K, Duthon VB, Menetrey J. Anterior cruciate ligament reconstruction using quadriceps tendon autograft is a viable option for small-statured female patients. Knee Surg Sports Traumatol Arthrosc. 2022 Jul;30(7):2358-63.

53. Ha JK, Kim JG, Yoon KH, Wang JH, Seon JK, Bae JH, Jang KM. Korean Version of the Anterior Cruciate Ligament-Return to Sport after Injury Scale: Translation and Cross-cultural Adaptation. Clin Orthop Surg. 2019 Jun;11(2):164-9.
54. Harput G, Tok D, Tunay VB. Relation of self-reported knee function and physical performance with psychological responses in anterior cruciate ligament reconstructed individuals. Fizyoterapi Rehabilitasyon. 2016;27(3):102-7.
55. Harput G, Tok D, Ulusoy B, Eraslan L, Yildiz TI, Turgut E, et al. Translation and cross-cultural adaptation of the anterior cruciate ligament-return to sport after injury (ACL-RSI) scale into Turkish. Knee Surg Sports Traumatol Arthrosc. 2017 Jan;25(1):159-64.
56. Hirohata K, Aizawa J, Furuya H, Mitomo S, Ohmi T, Ohji S, et al. The Japanese version of the anterior cruciate ligament-return to sport after injury (ACL-RSI) scale has acceptable validity and reliability. Knee Surg Sports Traumatol Arthrosc. 2020 Aug;28(8):2519-25.
57. Jia ZY, Cui J, Wang W, Xue CC, Liu TZ, Huang X, Xu WD. Translation and validation of the simplified Chinese version of the anterior cruciate ligament-return to sport after injury (ACL-RSI). Knee Surg Sports Traumatol Arthrosc. 2018 Oct;26(10):2997-3003.
58. Kim Y, Kubota M, Sato T, Inui T, Ohno R, Ishijima M. Psychological Patient-reported outcome measure after anterior cruciate ligament reconstruction: Evaluation of subcategory in ACL-Return to Sport after Injury (ACL-RSI) scale. Orthop Traumatol Surg Res. 2022 May;108(3):103141.
59. Kuenze C, Bell DR, Grindstaff TL, Lisee CM, Birchmeier T, Triplett A, Pietrosimone B. A Comparison of Psychological Readiness and Patient-Reported Function Between Sexes After Anterior Cruciate Ligament Reconstruction. J Athl Train. 2021 Feb 1;56(2):164-9.
60. Lee DW, Lee JK, Kwon SH, Moon SG, Cho SI, Chung SH, Kim JG. Adolescents show a lower healing rate of anterolateral ligament injury and a higher rotational laxity than adults after anterior cruciate ligament reconstruction. Knee. 2021 Jun;30:113-24.
61. McPherson AL, Feller JA, Hewett TE, Webster KE. Psychological Readiness to Return to Sport Is Associated With Second Anterior Cruciate Ligament Injuries. Am J Sports Med. 2019 Mar;47(4):857-62.
62. Phelan B, King E, Richter C, Webster K, Falvey E. A comparison of anterior cruciate ligament - Return to sports after injury (ACL-RSI) scores of male athletes nine-months Post-ACL reconstruction with matched uninjured controls. Phys Ther Sport. 2019 Jul;38:179-83.
63. Presley J, Bailey L, Maloney K, Duncan B, Reid M, Juneau C, Lowe WR. The Influence of Mode-of-Injury on Psychological Readiness for Return-To-Sport Following Anterior Cruciate Ligament Reconstruction: A Matched-Controlled Study. Int J Sports Phys Ther. 2021 Feb 1;16(1):177-84.
64. Rosso F, Bonasia DE, Cottino U, Cambursano S, Dettoni F, Rossi R. Factors Affecting Subjective and Objective Outcomes and Return to Play in Anterior Cruciate Ligament Reconstruction: A Retrospective Cohort Study. Joints. 2018 Mar;6(1):23-32.
65. Sadeqi M, Klouche S, Bohu Y, Herman S, Lefevre N, Gerometta A. Progression of the Psychological ACL-RSI Score and Return to Sport After Anterior Cruciate Ligament Reconstruction: A Prospective 2-Year Follow-up Study From the French Prospective Anterior Cruciate Ligament Reconstruction Cohort Study (FAST). Orthopaedic Journal of Sports Medicine. 2018;6(12):1-7.

66. Salatkaitė S, Šiupšinskas L, Žumbakytė-Šermukšnienė R, Gudas R. Eight-Months After Anterior Cruciate Ligament Reconstruction: Is It Time to Return to Physical Activity? Baltic Journal of Sport & Health Sciences. 2021;121(2):21-7.
67. Salatkaite S, Siupsinskas L, Gudas R. Translation and cultural adaptation of Lithuanian version of the anterior cruciate ligament return to sport after injury (ACL-RSI) scale. PLoS One. 2019;14(7):e0219593.
68. Slagers AJ, Reininga IH, van den Akker-Scheek I. The Dutch language anterior cruciate ligament return to sport after injury scale (ACL-RSI) - validity and reliability. J Sports Sci. 2017 Feb;35(4):393-401.
69. Slagers AJ, van den Akker-Scheek I, Geertzen JHB, Zwerver J, Reininga IHF. Responsiveness of the anterior cruciate ligament - Return to Sports after Injury (ACL-RSI) and Injury - Psychological Readiness to Return to Sport (I-PRRS) scales. J Sports Sci. 2019 Nov;37(21):2499-505.
70. Thiebat G, Cucchi D, Spreafico A, Muzzi S, Vigano M, Visconti L, et al. Italian version of the anterior cruciate ligament-return to sport after injury scale (IT ACL-RSI): translation, cross-cultural adaptation, validation and ability to predict the return to sport at medium-term follow-up in a population of sport patients. Knee Surg Sports Traumatol Arthrosc. 2022 Jan;30(1):270-9.
71. Tortoli E, Francini L, Giovannico G, Ramponi C. Translation, cross-cultural adaptation and validation of the Italian version of the anterior cruciate ligament-return to sport after injury (ACL-RSI) scale. Knee Surg Sports Traumatol Arthrosc. 2022 Apr;30(4):1180-6.
72. Webster KE, Feller JA. Evaluation of the Responsiveness of the Anterior Cruciate Ligament Return to Sport After Injury (ACL-RSI) Scale. Orthop J Sports Med. 2021 Aug;9(8):23259671211031240.
73. Welling W, Benjaminse A, Seil R, Lemmink K, Zaffagnini S, Gokeler A. Low rates of patients meeting return to sport criteria 9 months after anterior cruciate ligament reconstruction: a prospective longitudinal study. Knee Surgery, Sports Traumatology, Arthroscopy. 2018;26(12):3636-44.
74. Willson RG, Kostyun RO, Milewski MD, Nissen CW. Anterior Cruciate Ligament Reconstruction in Skeletally Immature Patients: Early Results Using a Hybrid Physeal-Sparing Technique. Orthop J Sports Med. 2018 Feb;6(2):2325967118755330.
75. Zanovello J, Rosso F, Bistolfi A, Rossi R, Castoldi F. Combined Intra- and Extra-Articular Technique in Revision Anterior Cruciate Ligament Reconstruction. Joints. 2017 Sep;5(3):156-63.
76. Zarzycki R, Arhos E, Failla M, Capin J, Smith AH, Snyder-Mackler L. Association of the Psychological Response to the ACL-SPORTS Training Program and Self-reported Function at 2 Years After Anterior Cruciate Ligament Reconstruction. Am J Sports Med. 2021 Nov;49(13):3495-501.
77. Zarzycki R, Failla M, Capin JJ, Snyder-Mackler L. Psychological Readiness to Return to Sport Is Associated With Knee Kinematic Asymmetry During Gait Following Anterior Cruciate Ligament Reconstruction. J Orthop Sports Phys Ther. 2018 Dec;48(12):968-73.
78. Meierbachtol A, Yungtum W, Paur E, Bottoms J, Chmielewski TL. Psychological and Functional Readiness for Sport Following Advanced Group Training in Patients With Anterior Cruciate Ligament Reconstruction. J Orthop Sports Phys Ther. 2018 Nov;48(11):864-72.

79. Muller U, Kruger-Franke M, Schmidt M, Rosemeyer B. Predictive parameters for return to pre-injury level of sport 6 months following anterior cruciate ligament reconstruction surgery. Knee Surg Sports Traumatol Arthrosc. 2015 Dec;23(12):3623-31.
80. Cusumano A, Capitani P, Messina C, de Girolamo L, Vigano M, Ravasio G, et al. Different timing in allograft and autograft maturation after primary anterior cruciate ligament reconstruction does not influence the clinical outcome at mid-long-term follow-up. Knee Surg Sports Traumatol Arthrosc. 2022 Jul;30(7):2281-90.
81. Sanborn RM, Badger GJ, Team BT, Yen YM, Murray MM, Christino MA, et al. Psychological Readiness to Return to Sport at 6 Months Is Higher After Bridge-Enhanced ACL Restoration Than Autograft ACL Reconstruction: Results of a Prospective Randomized Clinical Trial. Orthop J Sports Med. 2022 Feb;10(2):23259671211070542.
82. Rhim HC, Lee SJ, Jeon JS, Kim G, Lee KY, Lee JH, Jang KM. Effectiveness of modeling videos on psychological responses of patients following anterior cruciate ligament reconstruction: A pilot randomized trial. Medicine (Baltimore). 2020 Nov 6;99(45):e23158.
83. Ling DI, Chiaia TA, deMille P, Marx RG. Return to sport testing after anterior cruciate ligament reconstruction has marginal psychological impact: a randomised controlled trial. Journal of ISAKOS. 2019;4(1):4-8.
